# Pharmaceutical Formulations with P-Glycoprotein Inhibitory Effect as Promising Approaches for Enhancing Oral Drug Absorption and Bioavailability

**DOI:** 10.3390/pharmaceutics13071103

**Published:** 2021-07-20

**Authors:** Thi-Thao-Linh Nguyen, Van-An Duong, Han-Joo Maeng

**Affiliations:** College of Pharmacy, Gachon University, 191 Hambakmoe-ro, Yeonsu-gu, Incheon 21936, Korea; linh.nguyen@gachon.ac.kr (T.-T.-L.N.); anduong@gachon.ac.kr (V.-A.D.)

**Keywords:** P-gp, inhibitors, permeability, drug delivery, solid lipid nanoparticles, micelles, liposomes, polymeric nanoparticles, emulsions

## Abstract

P-glycoprotein (P-gp) is crucial in the active transport of various substrates with diverse structures out of cells, resulting in poor intestinal permeation and limited bioavailability following oral administration. P-gp inhibitors, including small molecule drugs, natural constituents, and pharmaceutically inert excipients, have been exploited to overcome P-gp efflux and enhance the oral absorption and bioavailability of many P-gp substrates. The co-administration of small molecule P-gp inhibitors with P-gp substrates can result in drug–drug interactions and increased side effects due to the pharmacological activity of these molecules. On the other hand, pharmaceutically inert excipients, including polymers, surfactants, and lipid-based excipients, are safe, pharmaceutically acceptable, and are not absorbed from the gut. Notably, they can be incorporated in pharmaceutical formulations to enhance drug solubility, absorption, and bioavailability due to the formulation itself and the P-gp inhibitory effects of the excipients. Different formulations with inherent P-gp inhibitory activity have been developed. These include micelles, emulsions, liposomes, solid lipid nanoparticles, polymeric nanoparticles, microspheres, dendrimers, and solid dispersions. They can bypass P-gp by different mechanisms related to their properties. In this review, we briefly introduce P-gp and P-gp inhibitors, and we extensively summarize the current development of oral drug delivery systems that can bypass and inhibit P-gp to improve the oral absorption and bioavailability of P-gp substrates. Since many drugs are limited by P-gp-mediated efflux, this review is helpful for designing suitable formulations of P-gp substrates to enhance their oral absorption and bioavailability.

## 1. Introduction

P-glycoprotein (P-gp) is a glycosylated membrane-bound protein that was discovered by Juliano and Ling in 1976 [1]. It is the adenosine triphosphate (ATP)-binding cassette sub-family B member 1 (ABCB1) and also known as multidrug resistance (MDR) protein. In 2018, Kim et al., reported the structure of human P-gp in an ATP-bound, outward-facing conformation using cryo-electron microscopy at a resolution of 3.4 angstroms [2]. P-gp is distributed throughout the body, including the intestine, liver, kidney, pancreas, brain, and placenta, and it actively transports many foreign substances out of cells [3]. Intestinal P-gp is present in the apical membrane of epithelial cells. Human P-gp is encoded by 28 exons in the MDR1/ABCB1 gene located on chromosome 7 at q^21^ [4]. Different single-nucleotide polymorphisms have been reported in the human MDR1 coding region and are attributed to single-nucleotide substitutions [5]. Human P-gp is a 1280-residue polypeptide comprised of two homologous halves and a flexible linker region. Mouse P-gp contains 1276 amino acids and shares 87% sequence identity with human P-gp [6]. Each half of P-gp has a transmembrane domain (TMD) and a nucleotide-binding domain (NBD) with a highly conserved ATP-binding site. The TMD is involved in drug recognition and transport, whereas the NBD is able to bind to ATP [7]. In the presence of adenosine triphosphatase (ATPase), ATP in the P-gp-ATP complex is hydrolyzed to adenosine diphosphate (ADP), releasing energy for active transport [8].

One of the most striking properties of P-gp is its ability to transport various substrates with diverse structures. These include analgesics (asimadoline, morphine), anticancer drugs (vinblastine, vincristine, paclitaxel (PTX), docetaxel, doxorubicin, daunorubicin, epirubicin, bisantrene, mitoxantrone, methotrexate, topotecan), human immunodeficiency virus (HIV) protease inhibitors (saquinavir, ritonavir, nelfinavir, indinavir, lopinavir, amprenavir), antiemetics (domperidone, ondansetron), corticoids (dexamethasone, hydrocortisone, corticosterone, triamcinolone), antibiotics (erythromycin, gramicidin D, valinomycin), compounds active against gout (colchicine) and diarrhea (loperamide), the calcium channel blocker verapamil, the cardiac glycoside digoxin, and immunosuppressive agents (cyclosporin A, FK506). P-gp substrates are usually amphipathic molecules with molecular weights (MWs) of 200–1900 Da; most contain aromatic groups that are uncharged or basic [9]. Therapeutic agents that are substrates of P-gp usually exhibit poor bioavailability or MDR activity [10,11]. P-gp can reduce the pharmacological response of drugs by impeding their permeability through physiological barriers [12]. Notably, P-gp alters drug absorption (e.g., by expelling drug molecules back into the gastrointestinal lumen in oral drug delivery) [13], distribution (e.g., by preventing drug penetration into the brain) [14], metabolism (e.g., by acting synergistically with cytochrome P450 3A) [15], and excretion (e.g., by affecting biliary and renal tubular functions) [12,13]. In addition, cells can exhibit cross-resistance to various drugs and cause MDR due to the broad substrate specificity of P-gp. P-gp is exclusively overexpressed in different types of tumors and transports many anticancer drugs, making the tumors resistant to such drugs [16]. 

The role of P-gp efflux in the pharmacokinetics of P-gp substrates has been increasingly appreciated [17]. It is well-known that in the human intestine, P-gp is highly expressed in epithelial cells of the colon and ileum (apical side). The expression level in the jejunum, duodenum, and stomach is relatively reduced compared to the ileum [18]. During oral absorption, drug properties (solubility and permeability) and P-gp efflux across the intestinal apical membrane determine the rate and amount of drug diffusing across the basolateral membrane to enter the general circulation [19]. Therefore, P-gp efflux screening is a key step in the drug discovery stage, aided by in vitro, in situ permeability studies and transgenic *mdr* knockout mice. It has been found that in vitro and in situ P-gp function for a given drug is well correlated with in vivo P-gp activity [17,20]. For example, Collett et al., revealed a positive quantitative relationship between P-gp-mediated changes in Caco-2 permeability (in the presence and absence of a P-gp inhibitor GF120918 (elacridar)) and P-gp effects on the oral bioavailability of ten P-gp substrates in wild-type and P-gp knockout mice [21]. The study was useful to develop models for the prediction of the in vivo pharmacokinetic properties from in vitro screens. In addition, effects of P-gp on the intestinal absorption and bioavailability of P-gp substrates have been reported in many previous studies. For example, by using wild-type mice and *mdr1a* gene-disrupted mice (*mdr1a*(−/−) mice), it was found that intestinal P-gp in mice substantially excreted [^3^H]-digoxin via gut epithelium [22]. In another study, Sparreboom et al., revealed the role of P-gp in limiting the oral uptake of PTX and mediating direct excretion of the drug from the systemic circulation into the intestinal lumen [23]. The data showed the increase in oral bioavailability of PTX from 11% in wild-type mice to 35% in *mdr1a*(−/−) mice and the decrease in the cumulative fecal excretion from 87% of the administered dose in wild-type mice to <3% in *mdr1a*(−/−) mice. In addition, Kim et al., found that P-gp limited the oral bioavailability and penetration into the brain of three HIV-1 protease inhibitors (indinavir, nelfinavir, and saquinavir) [24]. After oral administration, plasma concentrations at 4 h of three drugs in *mdr1a*(−/−) mice were 2−5-fold higher than wild-type mice. Similarly, the oral bioavailability of UK-224,671, a P-gp substrate, in *mdr1a*(−/−) mice was significantly higher than that in wild-type mice (22% versus < 2%) [25]. In another previous study, the AUC_0–5h_ of tacrolimus in *mdr1a*(−/−) mice was about 8-fold higher than in wild-type mice after oral administration [26]. Therefore, the P-gp efflux is considered one of the determining factors of oral bioavailability and intestinal efflux of drugs [18]. It is essential to overcome the absorption barrier caused by the P-gp, since the oral administration is one of the most important and preferable routes of drug delivery [19]. In addition, among lipophilic drugs in nature (about 50–60% of new and 40–50% of existing chemical entities), more than 25% are estimated to be P-gp substrates [27].

To overcome these problems, various P-gp inhibitors have been widely studied. They include small molecule drugs (active pharmaceutical ingredients (APIs) and new chemical entities (NCEs)), natural constituents, and pharmaceutically inert excipients [28,29,30]. In general, P-gp inhibitors can block drug-binding sites competitively, non-competitively, or allosterically, interfere with ATP hydrolysis, and alter the integrity of cell membranes [15]. In particular, for oral drug delivery, these inhibitors improve intestinal absorption, tissue distribution, and reduce substrate metabolism and elimination, resulting in enhanced pharmacokinetic properties and oral bioavailability [15]. For example, Mayer et al., revealed that the oral administration of PSC833 (valspodar) inhibited intestinal P-gp completely, which affected drug distribution and elimination [31]. Importantly, they found that the P-gp inhibitor reduced the direct excretion of [^3^H]-digoxin mediated by P-gp in the intestinal mucosa of wild-type mice. Another P-gp inhibitor, GF120918 (elacridar), was found to selectively and completely block intestinal P-gp function [32]. It increased the area under the curve (AUC) of oral PTX in wild-type mice by 6.6-fold, whereas it did not affect the pharmacokinetics of PTX in *mdr1a/1b* knockout mice (*mdr1a/1b*(−/−) mice), suggesting that the pharmacokinetics of PTX in wild-type mice after co-administration with GF120918 was comparable with those in *mdr1a/1b*(−/−) mice. Yang et al., demonstrated that 20(S)-ginsenoside Rh2 (Rh2s) was a good substrate of P-gp both in vitro and in vivo and that oral bioavailability of the compound was increased by a P-gp inhibitor (cyclosporine A) [33]. In *mdr1a/1b*(−/−) mice, the maximum plasma concentration (C_max_) and AUC_0–__∞_ of Rh2s were remarkably increased by 17- and 23-fold, respectively, compared with the wide-type mice. In the A/J mice (an inbred albino strain of mouse model), the oral bioavailability of Rh2s was considerably increased after co-administration with cyclosporine A. In a recent study, the AUC and C_max_ values of PTX after oral administration in *mdr1a/1b*(−/−) mice were 2.8−3.3 and 2.7–3.5-fold higher than those in wild-type mice, respectively [34], indicating that P-gp expressed in the intestinal epithelium restricted the intestinal absorption of PTX. In addition, the research group found that C_max_ and AUC values of PTX after oral administration in wide-type mice were significantly increased by pretreatment with LY335979 (zosuquidar), and similar to those in *mdr1a/1b*(−/−) mice, proving the critical impact of P-gp inhibitors in enhancing the intestinal absorption and oral bioavailability of P-gp substrates. Other studies also reported the effects of P-gp inhibitors in the improvement of intestinal absorption and oral bioavailability of P-gp substrates. The P-gp inhibitor PSC 833 enhanced the oral bioavailability of PTX in mice (10-fold) [35] and etoposide in rats [36]. Co-administration of the P-gp inhibitor HM30181 remarkably increased the oral bioavailability of PTX from 3.4% to 41.3% in rats [37]. GF120918 (elacridar) increased plasma concentrations of PTX (10.7-fold) and docetaxel (4-fold) [38]. The co-administration of [^14^C]-PTX with P-gp inhibitors, α-tocopheryl polyethylene glycol (PEG)-1000-succinate (TPGS) and verapamil, significantly increased the oral bioavailability of the compound 6.3- and 4.2-fold, respectively [39].

Small molecule P-gp inhibitors can be co-administered with P-gp substrates to enhance their oral bioavailability. However, this approach can result in drug–drug interactions and increased side effects because these small molecules have their own pharmacological activities. On the other hand, nonspecific P-gp inhibitors such as polymers, surfactants, and lipid-based excipients can be alternatives to these small molecules. They are safe, pharmaceutically acceptable, and are not absorbed by the gut [40]. They are incorporated in pharmaceutical formulations and indirectly inhibit P-gp by affecting the lipid membrane. Numerous formulations with inherent P-gp inhibitory activity have been studied for many years. These include micelles, emulsions, liposomes, microspheres, solid lipid nanoparticles, polymeric nanoparticles, dendrimers, and solid dispersions [41,42]. In addition to the P-gp inhibitory effect of the excipients, the formulations themselves can improve drug solubility and affinity to the intestinal membrane, promote paracellular passage and endocytic uptake, and enable the lymph transport pathway, resulting in improved oral bioavailability [43,44]. P-gp substrates with poor and inconsistent oral bioavailability due to poor permeability may have a marked benefit from combining with P-gp inhibitors. Namely, they are drugs in Biopharmaceutics Classification System (BCS) class III and IV, which have low permeability that limits drug absorption in the intestine. For example, P-gp inhibitors are useful to improve the permeation and bioavailability of many drugs in BCS class III, such as linagliptin [43], berberine [45], and gemcitabine [46], and BCS class IV, such as etoposide [47], docetaxel [48], PTX [49], and aripiprazole [50]. However, some drugs in BCS class II (low solubility and high permeability) are still beneficial from combining with P-gp inhibitors. Following oral administration, P-gp efflux may extrude them back to the intestinal tract, and P-gp inhibitors, such as verapamil and curcumin, increase their permeability in the intestine [51]. Some studies have reported the increased permeability of some BCS class II drugs after combining with P-gp inhibitors, such as irinotecan [52], candesartan [53], valsartan [54], and lovastatin [55]. Some P-gp substrate drugs, including diltiazem, quinidine, and verapamil, are in BCS class I with high solubility and high permeability. They are not required to combine with P-gp inhibitors because these drugs are usually used as P-gp inhibitors.

Several previous studies have reviewed nanocarriers for P-gp-mediated tumor drug resistance (i.e., cancer chemotherapy) [56,57,58,59,60], which are primarily used for parenteral administration. Since oral administration is one of the most important routes of drug delivery, various pharmaceutical formulations with P-gp inhibitory effects have been developed to enhance oral absorption and bioavailability of different P-gp substrates [41,42]. This review is the first publication to focus on the current development of different P-gp inhibitory formulations for the oral delivery of P-gp substrates. In this review, firstly, we present a brief introduction to P-gp, its efflux mechanism, and the classification of P-gp inhibitors, such as small molecules, natural products, and pharmaceutical excipients. Secondly, we summarize various formulation approaches with P-gp inhibitory activity, including emulsion, self-micro, and nano emulsifying drug delivery systems (SMEDDS, SNEDDS), liposomes, solid lipid nanoparticles (SLNs), nanostructured lipid carriers (NLCs), micelles, polymeric nanoparticles, microspheres, dendrimers, and solid dispersions. They can combine the P-gp inhibitory effects of their components and the ability to bypass P-gp efflux by different mechanisms attributed to the characteristic of each formulation [43,44]. Considering that many drugs are limited by P-gp-mediated efflux, this review could be helpful for designing the formulations of P-gp substrates.

## 2. Mechanism of P-gp Efflux and Functions of P-gp

### 2.1. Transport Cycles of P-gp Efflux

**Figure 1:**
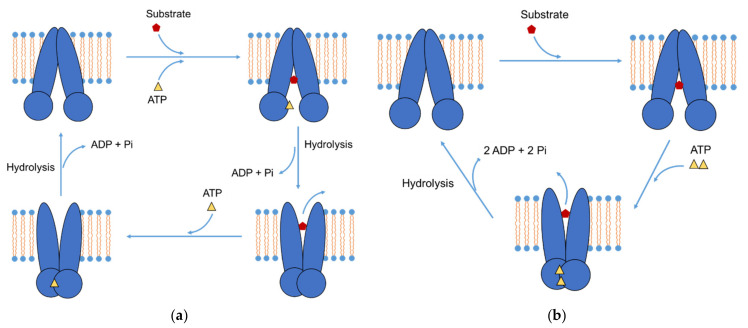
P-gp efflux mechanism. (**a**) “ATP hydrolysis as the power stroke” model and (**b**) “dimerization of the two ATP-binding sites as the power stroke” model describing the transport cycle of P-gp.

Several transport cycles of P-gp have been proposed to explain the efflux mechanism. Figure 1a shows the model of Sauna et al. [61,62,63] in which ATP hydrolysis was proposed to be the power stroke. In this transport cycle, P-gp binds to an ATP molecule and a substrate independently. Subsequent ATP hydrolysis changes the conformation of the drug-binding site to release the substrate. The conformation of NBDs is also changed, making them inaccessible to ATP. In the following steps, ADP and phosphate (Pi) are sequentially released to change the conformation of NBDs and make them accessible to ATP. After another ATP molecule is bound to P-gp, a second ATP hydrolysis event occurs followed by the subsequent release of Pi and ADP. P-gp is reset to the ground state, initiating a new transport cycle. 

Another model presented in Figure 1b proposed that the dimerization of the two ATP-binding sites was the power stroke [64]. In this model, a substrate binds to a binding site in the TMD followed by the binding of two ATP molecules to two NBDs. This dimerization changes the conformation of P-gp, which moves the substrate to an extracellular location and releases it. Next, the two ATP molecules are sequentially hydrolyzed, which provides the energy to release two ADP molecules and reset the P-gp to its ground state. 

The two models differ in the nature of the power stroke that drives the substrate from a high- to low-affinity site and releases it. The model in Figure 1a has two ATP hydrolysis events. One powers the substrate efflux and the other resets P-gp to the ground state. In the other model (Figure 1b), the substrate is released due to conformational changes after ATP binding, and two ATP hydrolysis events reset P-gp [65].

Recently, Kim et al., determined the structure of human P-gp in an ATP-bound, outward-facing conformation and proposed a model of the P-gp transport cycle similar to the model in which dimerization of the two ATP-binding sites is the power stroke [2]. The structure of human P-gp was stabilized by mutations that prevent ATP hydrolysis. In the observed structure, two NBDs formed a closed dimer, occluding two ATP molecules, whereas no substrate was bound to the substrate binding site in the TMD. The authors inferred that substrate release was promoted by ATP binding, not hydrolysis, and thereby proposed a model of P-gp transport similar to the model shown in Figure 1b. In the first step, P-gp is in the inward-facing state, and its two halves modulate the drug-binding cavity for substrate recruitment. The substrate subsequently enters the inner leaflet of the membrane due to the local conformational flexibility of P-gp. When P-gp binds two ATP molecules and isomerizes to the outward-facing state, the drug-binding site is rearranged and the substrate is released. P-gp is reset to its original inward-facing state after ATP hydrolysis [2].

### 2.2. Functions of P-gp in Drug Transport

P-gp is usually present in the apical membranes of epithelial cells. It transports substrates from the cell to the apical side and affects the pharmacological behavior of these substrates. P-gp functions are dependent on the location of P-gp [66]. For example, P-gp in the intestinal epithelium plays an essential role in reducing the oral bioavailability of drugs and toxins. It restricts the absorption of substrates from the intestinal lumen and decreases their blood concentration. In this case, P-gp is a major hindrance for the oral administration of drugs and limits their therapeutic applications [67]. Oral administration is much preferable in clinical use because it is relatively safe and convenient for patients. Low oral bioavailability indicates that the plasma level of the drug may be insufficient to produce therapeutic effects. Thus, in drug development, NCEs are usually tested to determine whether they are potent P-gp substrates. If they are, oral formulations containing them need to be carefully designed, and using P-gp inhibitors is one of the promising approaches [68]. 

The BBB is a highly selective, semipermeable border consisting of endothelial cells that are closely linked to each other and entirely cover the blood vessel wall. Compounds from the blood compartment can be transported into the brain by passive diffusion across the endothelial cell membranes. However, they are immediately pumped back (i.e., efflux) into the blood due to the activity of P-gp in the luminal membrane of endothelial cells [69]. The absence of functional P-gp in the BBB, such as in P-gp knockout mice, results in highly increased brain penetration of many drugs, which can cause neurotoxicity or alter their pharmacological effects. Thus, in the BBB, P-gp functions as a gatekeeper to protect the brain from toxins that are present in the blood. These toxins can be generated from food uptake or pathogenic organisms in the gastrointestinal tract [70]. 

P-gp is also present in the apical membrane of placental syncytiotrophoblasts, which are a specialized layer of epithelial cells. This layer is an essential barrier for nutrient and waste product exchange between maternal and fetal blood. Similar to P-gp, P-gp in the placenta protects the fetus from drugs and toxins present in the maternal blood [71]. In addition, P-gp is present on the bile canalicular membrane of hepatocytes, the apical surface of epithelial cells of small bile ducts, and the upper surface of epithelial cells of the proximal convoluted tubules of the kidney. Its role is to eliminate drugs and toxins through the urine and bile [72].

P-gp inhibitors are often required to reduce the activity of P-gp. For oral drug administration, inhibiting P-gp can increase drug absorption and bioavailability and thus its therapeutic effects [49]. P-gp inhibition in the BBB can increase the transport of some brain-targeted drugs through the BBB and improve its effects in the brain [73]. In the case of an HIV treatment, P-gp inhibitors are useful to increase drug loading of the fetus shortly before birth, which can reduce the frequency of HIV transmission during birth [9]. P-gp inhibitors can reverse the MDR phenomenon associated with P-gp efflux, resulting in the improved efficiency of chemotherapeutic agents and enhanced pharmacokinetic and pharmacodynamic profiles of drugs. 

### 2.3. Experimental Studies for Evaluation of P-gp Efflux

In the oral drug delivery, some models have been used to determine the drug permeability and evaluate the P-gp efflux, such as Caco-2 or Madin Darby canine kidney (MDCK)-MDR1 cell monolayers, inside-out vesicles, single-pass intestinal perfusion (SPIP), and everted gut sac permeability studies. P-gp efflux can be determined by comparing the permeability of a drug between a test sample (a formulation) and a control sample (usually a drug solution).

#### 2.3.1. Caco-2, MDCK-MDR1 Cell Monolayers, and Inside-Out Vesicles

Caco-2 cells derived from human colon carcinoma cells, which express P-gp and metabolizing enzymes, such as peptidases, are widely used as an in vitro model of human small intestinal mucosa to predict the absorption of orally administered drugs [74,75,76]. Caco-2 cell monolayers are usually cultured on semipermeable plastic supports that may be fitted into wells of multi-well culture plates. Then, the test and control samples are added to either the apical or basolateral sides of the monolayer. After incubation for various lengths of time, aliquots of the buffer in opposite chambers are removed for analysis and calculation of drug permeability in formulations or free form [77,78]. The apparent permeability coefficient (P_app_) and efflux ratio (ER) are determined as follows [79,80]:

(1) $$P_{\mathrm{app}}=\frac{\mathrm{dQ}}{\mathrm{dt}}\times \frac{1}{A\times C_{0}}$$

(2) $$\mathrm{ER}=\frac{P_{\mathrm{app}\;\left(B-\mathrm{to}-A\right)}}{P_{\mathrm{app}\;\left(A-\mathrm{to}-B\right)}}$$

where dQ/dt is the permeability rate, A is the effective surface area, C_0_ is the initial concentration of the drug, P_app (B-to-A)_ is the apparent permeability of the drug in the basolateral-to-apical direction, and P_app (A-to-B)_ is the permeability in the apical-to-basolateral direction. 

MDCK-MDR1 cells are MDCK cells transfected with MDR1 to overexpress P-gp [81]. Similar to Caco-2 cells, they are usually used to evaluate drug permeability and efflux mediated by P-gp [82,83]. MDCK-MDR1 cells are also useful for investigating drug permeability across the BBB. Inside-out vesicles are produced from plasma membranes with their cytoplasmic face oriented outward. When expressing human MDR1 transporters, inside-out vesicles can be used to study drug excretion by P-gp [84]. The transport direction is from outside-to-inside of the vesicles, instead of across the cell monolayers, as in Caco-2 and MDCK-MDR1 cell models [85].

Other studies evaluated P-gp efflux using the Chemicon^®^ MDR dye efflux assay kit [86,87]. In these analyses, the intracellular accumulation of fluorescent dyes, such as Rhodamine 123 and 3,3′-diethyloxacarbocyanine (DiOC_2_) is used to assess the inhibitory effect of drug solutions and drug-loaded formulations on P-gp transporters. Caco-2 cells are incubated with the samples, and fluorescence intensity is measured in the presence of dye-loaded formulations.

#### 2.3.2. SPIP

The in situ SPIP method has been used to evaluate the permeability of drugs in different intestinal segments. The rat intestine is frequently used in this model. In this method, after anesthesia, the rat abdomen is opened, and an intestinal segment (duodenum, jejunum, ileum, or colon) is exposed and cannulated at both ends. The isolated segment is washed, and a drug-free perfusion solution (blank perfusion solution) at 37 °C is pumped through the intestine using a peristaltic pump to remove residual debris. Subsequently, a perfusion solution containing the drug at 37 °C is perfused at a constant flow rate through the intestinal lumen for equilibration. After a steady state, samples are collected from the distal portion of the intestinal segment at different time points for analysis (usually every 15 min for a total of 120 min) [88]. 

The absorption rate (K_a_) and effective permeability coefficient (P_eff_) of a drug are calculated as follows [88,89]:

(3) $$K_{a}=\left(1-\frac{C_{\mathrm{out}}}{C_{\mathrm{in}}}\times \frac{V_{\mathrm{out}}}{V_{\mathrm{in}}}\right)\times \frac{Q}{V}$$

(4) $$P_{\mathrm{eff}}=-\frac{Q_{\mathrm{in}}}{A}\times \ln\left(\frac{C_{\mathrm{out}}}{C_{\mathrm{in}}}\times \frac{Q_{\mathrm{out}}}{Q_{\mathrm{in}}}\right)$$

where C_out_ and C_in_ are the outlet and inlet drug concentration, respectively; Q_out_ and Q_in_ are the outlet and inlet perfusate flux (mL/min), respectively; and *V* and *A* are the intestinal volume and area for absorption, respectively. 

This model has been used to evaluate the drug permeability of different drug delivery systems, such as emulsions [79], self-microemulsifying drug delivery systems (SMEDDS) [54], liposomes [90], and nanoparticles [91]. Some studies used P_app_, which was also calculated using Equation (4) [47,48].

#### 2.3.3. Everted Gut Sac Permeability Studies

The everted gut sac of the rat intestine model was first introduced in 1954. It has become a widely employed paradigm for the determination of drug absorption kinetics and evaluation of the effects of absorption enhancers [92,93,94]. To prepare the everted gut sac, the jejunum, duodenum, or ileum of the intestine are removed and divided into 5–6 cm long segments. The segments are washed and everted over a glass rod. One end of the intestine is clamped, and the intestine is usually filled with Krebs–Ringer solution. Then, the sealed intestine in the acceptor compartment (serosal) is transferred to the incubation flask containing the drug (donor compartment), and sampling is performed at different time intervals. The drug concentration in the acceptor compartment is determined by calculating the apparent permeability coefficient. Although in vitro and in vivo correlations between the everted sac and in vivo models have not been established, frequently obtained results from the everted intestinal sac model have agreed with in vivo findings [95]. Therefore, this model has been extensively explored with modifications and improvements to perform pharmacokinetic investigations that have included drug absorption, drug metabolism, MDR, efflux transport, and the impact of efflux transport modulators on drug absorption. The advantages of this model are the relatively large surface area available for absorption and the presence of a mucus layer. However, tissue viability is a limitation. Several animals have been chosen for everted gut sac models. The everted rat intestinal sac is most commonly used. For example, the rat everted gut sac was used to investigate the P-gp-mediated efflux of various anticancer agents, such as vinblastine, doxorubicin, and etoposide [96,97]. The everted gut sac model has also been used to investigate the role of pharmaceutical excipients and formulations in transport modulation. Surfactants, such as Cremophor EL, polysorbate 80 [98], Pluronic F68 [99], and TPGS [97] and formulations that include cyclodextrin inclusion complexes [100], nanoparticles [101], mixed-micellar proliposomal systems [102], nanodispersions [103], and solid lipid nanoparticles [43] of P-gp substrates can be screened using the everted sac model. The reproducible results of this in vitro model suggest its usefulness for transport studies of P-gp substrates and potential P-gp inhibitors. In everted gut sac permeability studies, P_app_ can be calculated using Equation (1) [104].

## 3. Classification of P-gp Inhibitors

P-gp inhibitors are compounds that block or bypass P-gp efflux. The concurrent administration of P-gp inhibitors with P-gp substrates can prevent the expulsion of these substrates and increase their therapeutic effects. Researchers have identified, studied, and evaluated various P-gp inhibitors, including small molecules, natural products, and pharmaceutically inert excipients [60,105]. Additionally, prodrugs, synthetic peptides, and P-gp expression suppressors are also potential approaches to overcome P-gp efflux [106]. 

### 3.1. Small Molecules

Small molecules that include APIs and NCEs are usually considered specific P-gp inhibitors. They can be co-administered with a P-gp substrate to effectively enhance oral bioavailability. However, these compounds have their own pharmacological activity, which can result in potential drug–drug interactions and increased side effects. Cross-interaction of the inhibitors with P-gp in other organs/tissues is another issue to consider as shown in the new generation of P-gp inhibitors [107]. These can be classified into three generations. The first-generation inhibitors were primarily developed for other indications but were later identified as P-gp substrates and P-gp inhibitors. Being P-gp substrates, they may interact with P-gp, compete with other substrates, and act as competitive inhibitors. Thus, they are usually less potent, nonselective, and have unwanted side effects [108]. Many of these inhibitors failed in clinical trials because their P-gp inhibitory levels were as high as their toxic concentrations [107]. First-generation compounds include verapamil (an antihypertensive calcium channel blocker), cyclosporine (an immunosuppressant), and trifluoperazine (a calmodulin antagonist). Other APIs that are first-generation inhibitors include quinidine, reserpine, yohimbine, tamoxifen, toremifene, and vincristine [109]. 

The structure of some inhibitors in the first generation was modified to reduce their toxicity. These second-generation P-gp inhibitors may have no pharmacological effects. An example is dexverapamil, an R-isomer of verapamil with no cardiac activity. Another example is PSC 833 (valspodar), a cyclosporine A analog with no immunosuppressive activity. They are still P-gp substrates with low protein affinity and are used at a P-gp inhibitory dose that greatly exceeds the tolerable doses. These inhibitors are also substrates of cytochrome P450 3A4 due to chiral optimization. Thus, they may compete with other anticancer drugs, causing significant pharmacokinetic alterations and unpredicted metabolic and clearance mechanisms of these drugs [110,111].

Finally, third-generation inhibitors, such as VX-710 (biricodar), GF120918 (elacridar), LY335979 (zosuquidar), XR9576 (tariquidar), WK-X-34, and OC144-093 (ontogeny), were developed with greater potency and selectivity. Compared with the first and second-generation inhibitors, they can inhibit P-gp efflux activity at lower concentrations. Additionally, they have a lower affinity for CYP3A4 than the second-generation inhibitors. Third-generation inhibitors can inhibit P-gp at a low concentration (30–100 nM) and are at least 10-fold more potent than earlier inhibitors [107]. A recent study compared different P-gp inhibitors and found that LY335979 could be the most valuable inhibitor for investigations of P-gp contribution to drug absorption [34]. Novel P-gp inhibitors are still being developed and investigated [29,112,113,114].

### 3.2. Natural Products

Various natural bioactive constituents with P-gp-modulating effects have been reported. They are sometimes considered fourth-generation inhibitors of P-gp because of their low toxicity. This group includes alkaloids (glaucine, pervilleine, berberine, kopsiflorine, lobeline, cepharanthine, ibogaine, theobromine), flavonoids (quercetin, morin, phloretin, rhamnetin, plagiochin E, daidzin, procyanidine, rotenone), coumarins (decursinol, bergaptol, galbanic acid, farnesiferol), terpenoids (citral, latilagascene, paraliane, pepluanin A, jolkinol B, euphoportlandol, helioscopinolide, tuckeyanols, euphotuckeyanol, isopimaric acid, totarol), saponins (gracillin, tenacissimoside A, karavilagenin C, balsaminol, ginsenoside F1, protopanaxatriol), peptides (discodermolide, kendarimide, hapalosin, nocardioazine), resins (gambogic acid, orizabin), and miscellaneous natural compounds (acetoxy chavicol acetate, arctigenin, pheophorbide, porphyrin, cannabinol, gomisin, pregomisin, phenylbutanoid). Structures, mechanisms of action, and inhibitory concentrations of more than 250 natural P-gp inhibitors have been summarized in a previous review [72]. Natural products can be modified by semi-synthesis to improve their activities. Generally, products of natural origin are considered safe and less toxic. Thus, natural P-gp inhibitors have attracted research interest. However, they may have unwanted pharmacokinetic changes [115] and toxicities due to the non-specificity for P-gp [72]. The presence of constituents with P-gp inhibitory effects in fruits, vegetables, plant products, and medicinal herbal preparations may cause some unexpected effects when they are concomitantly administered with P-gp substrates. Therefore, food–drug, plant–drug, and herbal medicine–drug interactions should be considered in these cases. More research is required to investigate these natural products by identifying their optimized inhibitory concentrations, pharmacokinetic properties, potential toxicities, and possible drug–drug interactions [116].

### 3.3. Pharmaceutical Excipients

Pharmaceutical excipients can interact with lipid bilayers and indirectly or nonspecifically modulate P-gp activity. Generally, pharmaceutical excipients exhibit a low nonspecific pharmacological activity and thus do not cause serious side effects compared with APIs and NCEs. They are usually surface-active compounds and are considered to be nonspecific inhibitors of P-gp. These excipients can bind to the non-transporting active site on P-gp, change the protein conformation, and alter the integrity of the cell membrane, which results in P-gp dysfunction. For example, TPGS binds to the ATP-binding sites of NBDs and subsequently reduces substrate efflux [117,118]. Amphiphilic pluronic block copolymers reduce membrane microviscosity and increase intestinal membrane permeability. The polymers also cause cellular depletion of ATP levels and membrane permeability changes through a reduction in membrane microviscosity [119]. These pharmaceutical excipients include surfactants, polymers, and other lipid-based excipients. In addition to indirect P-gp inhibition, the presence of these excipients in pharmaceutical formulations can also increase drug solubility and stability and thereby increase drug absorption and bioavailability.

Surfactants that can inhibit P-gp consist of polysorbates (e.g., polysorbate 80, 20), sucrose esters (e.g., sucrose monolaurate), tocopheryl esters (e.g., TPGS), PEG esters (e.g., PEG-35 castor oil; Cremophor EL), PEG-15-hydoxystearate (Solutol HS-15), PEG-8 caprylic/capric glycerides (Labrasol), PEG-6 caprylic/capric glycerides (Softigen 767), PEG stearate (Myrj), PEG-32 laurate (Gelucire 44/14), PEG ethers (PEG-20 cetyl ether (Brij 78)) [40,60], sodium 1,4-bis (2-ethylhexoxy)-1,4-dioxobutane-2-sulfonate (AOT), and cetyltrimethylammonium bromide (CTAB) [120]. A typical example of these surfactants is TPGS, which is an amphiphilic surfactant that can be co-administered with a P-gp substrate to enhance drug solubility, inhibit P-gp efflux, and improve oral bioavailability [56]. P-gp inhibition may be attributed to alterations in membrane fluidization, the blockage of substrate binding sites, and allosteric modulation of P-gp [121]. This surfactant is also widely used to stabilize nanoparticulate drug delivery systems [122]. 

Typical polymers used as P-gp inhibitors are natural polymers (e.g., dextrans, agar, gellan gum, gum arabic, gum traganth, guar gum, carrageenan gum, xanthan gum, alginates, chitosan) [123], PEG, amphiphilic copolymers (e.g., methoxyPEG-block-polycaprolactone, polyvinyl caprolactam-polyvinyl acetate-PEG graft copolymer (Soluplus), and Pluronic block copolymers (poloxamer) [60,124]. Poloxamers are triblock copolymers consisting of hydrophilic poly(ethylene oxide) (PEO) and hydrophobic poly(propylene oxide) (PPO) in a PEO-PPO-PEO structure. For example, poloxamer 407 is PEO101-PPO56-PEO101, whereas poloxamer 188 was PEO75-85-PPO25-30-PEO75-85. Poloxamers can inhibit P-gp by depleting ATP, inhibiting ATPase, and modifying membrane fluidization. They have been widely used for the preparation of polymeric micelles, nanoparticles, and liposomes [125]. Soluplus is an amphiphilic graft copolymer with a low critical micelle concentration (CMC). It has been used as a component of liposomes [126], micelles [127], and solid dispersions [128].

Some lipid-based excipients have P-gp inhibitory effects, such as glycerides (e.g., monoolein (Peceol^TM^) and monostearin) [129,130] and phospholipids (e.g., 1,2-dioctanoyl-sn-glycero-3-phosphocholine (8:0 PC) and 1,2-didecanoyl-sn-glycero-3-phosphocholine (10:0 PC)) [131]. In addition, methylated cyclodextrin can inhibit P-gp by interacting with the lipid components of biological membranes, which modifies their fluidity and permeability [132]. A summary of the P-gp inhibitors is presented in Table 1.

## 4. Recently Developed Formulations to Bypass P-gp and Enhance Oral Bioavailability of P-gp Substrates

Some drug delivery systems have emerged as promising approaches to bypass and inhibit P-gp efflux. These include micelles, liposomes, emulsions, SMEDDS, SNEDDS, SLNs, NLCs, polymeric nanoparticles, microspheres, dendrimers, and solid dispersions. These drug delivery systems can combine the P-gp inhibitory effects of their components and the ability to bypass P-gp efflux by themselves [43,44]. Two major approaches are frequently used for the incorporation of P-gp inhibitors: (i) co-administration of a small drug molecule as a specific P-gp inhibitor or a natural constituent and (ii) co-encapsulation of both P-gp substrates and P-gp inhibitors in a drug delivery system [30]. In the latter case, the P-gp inhibitor can be a small drug molecule, natural constituent, pharmaceutical excipient, or combinations of these [44,133,134]. 

**Figure 2:**
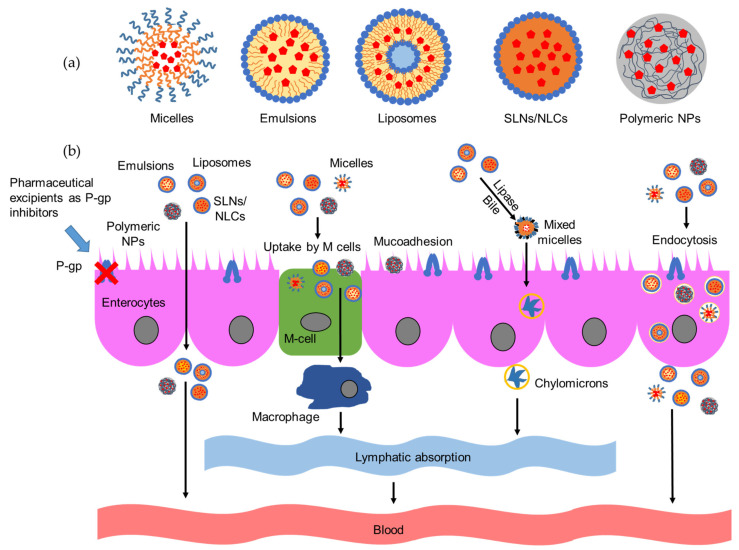
Drug delivery systems can bypass and inhibit P-gp efflux by various effects. (**a**) Typical drug delivery systems to encapsulate P-gp substrate to enhance oral bioavailability. (**b**) Pharmaceutical excipients as components of various drug delivery systems can inhibit P-gp efflux. Different effects of nano drug delivery systems to enhance drug absorption and oral bioavailability. SLNs: solid lipid nanoparticles; NLCs: nanostructured lipid carriers; NPs: nanoparticles.

In addition to P-gp inhibitory effects due to the presence of P-gp inhibitors in their components, which result in increased intestinal absorption and bioavailability of P-gp substrates, these drug delivery systems, particularly nanocarriers, can also enhance oral bioavailability by various effects, as shown in Figure 2 [135]. First, they increase drug solubility, dissolution, and affinity to the intestinal membrane, leading to an increase in drug absorption by enterocytes [28]. This effect is common for most oral drug delivery systems, particularly nanocarriers, which have large specific surface areas. Most drug delivery systems can also protect drugs from degradation in the gastrointestinal tract [136]. Second, some formulations can improve mucoadhesion due to the interaction between the nanocarrier (positive charge) and mucin (negative charge). For example, chitosan and carboxymethyl chitosan have strong mucoadhesive properties and can be used to prepare micelles with enhanced mucoadhesion [44,137]. 

Third, nanocarriers may interact with tight junction proteins and modulate tight junctions in the intestinal epithelium, resulting in paracellular transport [89,138]. The extent of tight junctions is generally evaluated by measuring the transepithelial electrical resistance (TEER) of Caco-2 monolayers. An increase in paracellular permeability is indicated by a decrease in TEER. SMEDDS containing Labrasol [139], chitosan nanoparticles [140], *N*-trimethyl chitosan nanoparticles [141], carboxymethyl chitosan micelles [44], and NLCs [142] can transiently widen tight junctions to enhance intestinal absorption. 

Fourth, receptor-mediated endocytosis and the transcytosis of enterocytes can enhance the transcellular transport of nanocarriers [143]. Particles <500 nm in size can be absorbed by intestinal enterocytes and bypass P-gp efflux, making this route an essential transport pathway for nanocarriers [144]. The endocytosis mechanisms of drug delivery systems can be explored by using the everted intestinal ring model. In this model, different endocytosis inhibitors are used, including indomethacin and nystatin (caveolae-dependent endocytosis inhibitors), chlorpromazine (clathrin-dependent endocytosis inhibitor), quercetin (caveolae- and clathrin-independent endocytosis inhibitor), colchicine and amiloride (micropinocytosis inhibitors), methyl-β-cyclodextrin (cholesterol-dependent endocytosis inhibitor), and sodium azide (inhibitor of energy-dependent transport) [53,91,133]. Decreased drug permeability after the addition of an endocytosis inhibitor suggests a corresponding endocytosis mechanism for the drug delivery systems. Zhang et al., developed super-antiresistant micelles that were transported across Caco-2 cell monolayers via caveolae-mediated endocytosis, micropinocytosis, cholesterol-dependent endocytosis, and clathrin-mediated endocytosis [145]. 

Fifth, some nanocarriers, such as SLNs, NLCs [135], polymer–lipid hybrid nanoparticles (PLHNs) [146], and micelles [44], can be phagocytosed by microfold cells (M cells). These are specialized cells in the lymphoid tissue of Peyer’s patches in the small intestine. In other parts of the gastrointestinal tract, M cells are located in the mucosa-associated lymphoid tissue. They allow the transport of microbes and small particles across the epithelial cell layer. After the selective endocytosis of antigens, M cells transport them to intraepithelial macrophages and lymphocytes prior to their migration to lymph nodes [147]. Thus, the drug can bypass the P-gp efflux and liver metabolism. Another effect that occurs with various lipid-based drug delivery systems is lymphatic absorption from enterocytes (mediated by lipase and chylomicron uptake). It is one of the major absorption mechanisms for drugs encapsulated in SLNs, NLCs, emulsions, and liposomes [148,149]. Lipase digests triglycerides in the stomach to form crude emulsified lipid systems. They are subsequently exposed to bile salts, cholesterol, and pancreatic lipase and further digested to form mixed micelles of the lipidic system containing drugs. Then, these micelles are absorbed by enterocytes and converted to chylomicrons, which can be transported via the mesenteric lymph to enter the lymphatic transport system. The chylomicrons ultimately enter the systemic circulation via lymphatic drainage at the thoracic duct [150]. 

Different formulations have been developed and employed to enhance the oral absorption and bioavailability of various P-gp substrates. The following sections describe the recent development of pharmaceutical formulations for the oral delivery of a wide range of P-gp substrates.

### 4.1. Emulsion, Self-Micro, and Nano Emulsifying Drug Delivery Systems

Emulsions are colloidal systems prepared from two immiscible liquids with force and the addition of surfactants, in which one phase is dispersed as tiny droplets in the other phase (Figure 2a) [151]. The dispersed phase is also called the discontinuous phase or internal phase. The outer phase is also termed as the continuous phase, external phase, or dispersion medium. The surfactants have affinities for both phases and are located on the interfacial surface of the two liquids to stabilize the emulsions. Two types of emulsions are oil-in-water and water-in-oil emulsions, depending on the volume ratio of the two liquids. Methods used to prepare emulsions include ultrasonication, high-pressure homogenization, microfluidization, phase inversion temperature, and spontaneous emulsification [152]. SMEDDS and SNEDDS are lipid-based nanocarriers that spontaneously emulsify to form fine oil-in-water emulsions after exposure to gastrointestinal fluids. SMEDDS and SNEDDS are stable mixtures of oil, surfactants, and cosurfactants. SMEDDS contain a lower percentage of lipids and a higher percentage of hydrophilic surfactants and cosurfactants compared with SNEDDS. SMEDDS usually form thermodynamically stable emulsions <100 nm in size. Emulsions derived from SNEDDS range in size from 100 to 250 nm [153]. Emulsions, SMEDDS, and SNEDDS have been widely used as drug delivery systems to enhance the oral absorption and bioavailability of various lipophilic drugs [154,155]. These benefits are attributed to the endocytosis of enterocytes, paracellular transport, and lymphatic absorption via chylomicron uptake. In addition, the lipids and surfactants used for the preparation of emulsions, SMEDDS, and SNEDDS may have P-gp inhibitory effects, which considerably reduce P-gp efflux and increase the absorption of P-gp substrates.

For example, a nanoemulsion was prepared to encapsulate paeonol using isopropyl myristate as oil, Cremophor EL35 as a surfactant, and ethanol as a cosurfactant [79]. Paeonol is a phenolic compound found in peonies, which has anti-inflammatory, antibacterial, and antitumor activities. Its clinical applications have been hampered, owing to its poor water solubility, P-gp efflux, poor stability, and low oral bioavailability. In Caco-2 cell monolayers, paeonol nanoemulsions increased P_app (A-to-B)_ by 1.7-fold and decreased ER by 1.7-fold compared with the paeonol solution. However, the addition of verapamil did not affect P_app (A-to-B)_, P_app (B-to-A)_, or ER of the paeonol nanoemulsion. A SPIP study in rats revealed an approximately 1.5-fold increase in P_app_ for paeonol nanoemulsions in the ileum and colon compared with the paeonol solution. In the in vitro everted rat gut sacs, the nanoemulsion improved drug permeability (P_app_) by approximately 2-fold, whereas the co-administration of verapamil caused no further enhancement. These findings were similar to those of the Caco-2 cell monolayer study, indicating that the nanoemulsion could protect paeonol from being identified and discharged by P-gp. The authors also revealed that Cremophor EL35 was a P-gp inhibitor, as evidenced by the increased P_app_ of paeonol when dissolved in Cremophor EL35 solution. The P_app_ of the nanoemulsion was approximately 1.5-fold higher than that of the drug solution in Cremophor EL35, suggesting that the nanoemulsion could enhance drug permeability by a method other than the P-gp inhibitory effect of the surfactant. A pharmacokinetic analysis in rats revealed an increase in oral bioavailability (4.27-fold) and C_max_ (4.02-fold) of the paeonol nanoemulsion compared with the drug suspension. 

In addition to emulsion, SMEDDS and SNEDDS were used to load many P-gp substrates. Etoposide is an alkaloid used for the treatment of different types of cancer, including lymphoma, leukemia, lung cancer, ovarian cancer, and neuroblastoma. Etoposide is a P-gp and cytochrome P450 3A substrate with poor aqueous solubility. Zhao et al., prepared three SMEDDS formulations containing known P-gp inhibitory surfactants: Cremophor RH40, Cremophor EL, and polysorbate 80 [47]. Among them, polysorbate 80-based SMEDDS showed the most significant increase in etoposide solubility and Caco-2 cellular uptake. In an SPIP study using rat ileum, polysorbate 80-based SMEDDS demonstrated the highest increase in drug permeability (2.7-fold). The bioavailability of etoposide from Cremophor RH40-, Cremophor EL-, and polysorbate 80-based SMEDDS was increased by 1.4-, 1.7-, and 2.5-fold, respectively, compared with that of the etoposide suspension after oral administration in rats. The authors also found a high linear correlation between apparent permeability coefficient values in SPIP and oral bioavailability data, suggesting that the enhanced bioavailability was attributed to the improved drug permeability.

Negi et al., developed SMEDDS loaded with irinotecan, which is a camptothecin derivative with low oral bioavailability due to P-gp efflux [52]. The SMEDDS were prepared from Capmul MCM-C8 (oil), Cremophor EL (surfactant), and Pluronic L-121 (cosurfactant). The SMEDDS spontaneously produced nanoemulsions of particles (approximately 130 nm in size). These SMEDDS penetrated deeper into the intestine as revealed by confocal laser scanning microscopy and displayed greater uptake of fluorescent probe in Caco-2 cells, as indicated by flow cytometry. In a pharmacokinetic study in rats, these SMEDDS increased oral bioavailability by 4.29-fold and C_max_ by 1.77-fold compared with the drug suspension. In addition, the SMEDDS showed a sustained release of irinotecan with a prolonged half-life (t_1/2_). Thus, these SMEDDS could effectively modulate P-gp efflux and increase the oral absorption of irinotecan.

In another study, candesartan cilexetil-loaded SNEDDS was developed using peppermint oil, Cremophor RH40, and Labrasol [53]. Candesartan cilexetil is a prodrug of candesartan, which is a selective antagonist of the angiotensin II subtype-1 receptor that is widely used in the management of heart failure and hypertension. Poor aqueous solubility and P-gp efflux result in low oral bioavailability, which limits its clinical use. In the everted gut sac permeability studies, SNEDDS increased the P_app (A-to-B)_ of the drug by approximately 2- and 1.74-fold compared with drug solution and drug + verapamil solution, respectively. The ER of SNEDDS was 3.52-fold lower than that of the drug solution, indicating that the drug-loaded SNEDDS could inhibit P-gp efflux and enhance drug absorption. The authors also determined the main endocytosis pathways for candesartan cilexetil-loaded emulsions (formed after dispersing SNEDDS in the Krebs–Ringer buffer solution), which included caveolae-dependent endocytosis, caveolae-independent endocytosis, clathrin-independent endocytosis, and micropinocytosis pathways. In a pharmacokinetic study in rabbits, SNEDDS showed higher oral bioavailability (1.69-fold) and C_max_ (1.75-fold) than a brand product of candesartan (ATACAND tablets). The enhanced bioavailability observed in this study was due to the multiple functions of SNEDDS, such as endocytosis of ultrafine oil droplets and P-gp inhibitory effect of Cremophor RH40 and Labrasol. 

Cui et al., co-loaded docetaxel and cyclosporine A in SNEDDS using Capryol 90, Cremophor EL, and Transcutol HP [48]. Docetaxel is a taxane-based anticancer drug that has been widely used for the treatment of various cancers. It has a very low oral bioavailability due to enzymatic degradation in the gastrointestinal tract, poor aqueous solubility, low intestinal permeability, P-gp efflux, and rapid hepatic first-pass metabolism. In an SPIP study using rat intestine, the absorption rate (K_a_) and apparent permeability (P_app_) were highest in the drug cyclosporine A co-loaded SNEDDS, followed by the drug-loaded SNEDDS and the drug solution in four intestinal segments (duodenum, jejunum, ileum, and colon). SNEDDs increased the K_a_ and P_app_ of docetaxel by approximately 1.5-fold, whereas co-encapsulation of cyclosporine A into the SNEDDS further enhanced these values by approximately 2-fold. The oral bioavailability of docetaxel–cyclosporine A co-loaded SNEDDS was 9.2- and 3.4-fold higher than that of the drug solution and drug-loaded SNEDDS, respectively. Additionally, in tumor-bearing mice, docetaxel-loaded SNEDDS and docetaxel–cyclosporine A co-loaded SNEDDS showed enhanced antitumor efficiency compared with docetaxel solution after oral administration. Notably, oral co-loaded SNEDDS reduced tumor volumes, similar to the intravenous docetaxel solution. Thus, the co-encapsulation of a specific P-gp inhibitor (cyclosporine A) and a P-gp substrate (docetaxel) in SNEDDS could facilitate oral chemotherapy by improving drug absorption and bioavailability. In addition to the P-gp inhibitory effect of cyclosporine A, Cremophor EL also inhibited P-gp, whereas SNEDDS increased drug absorption by other effects, such as endocytosis of emulsion, paracellular transport, M cell uptake, and lymphatic absorption via chylomicron uptake.

Recently, Goo et al., developed a supersaturable SMEDDS (Su-SMEDDS) with Capmul MCM as oil, Transcutol P as a cosurfactant, Poloxamer 407 as a supersaturating agent, and polysorbate 20, polysorbate 80, Cremophor EL, and Labrasol as surfactants [54]. The Su-SMEDDS encapsulated valsartan, a selective inhibitor of angiotensin II receptor type 1, is widely used in the treatment of hypertension and congestive heart failure. It has poor aqueous solubility and low permeability in the gastrointestinal tract, which results in low oral bioavailability. In an SPIP study using rat jejunum, the P_eff_ of Su-SMEDDS prepared with polysorbate 20, Cremophor EL, and polysorbate 80 were 2.3-, 3.4-, and 4.1-fold higher than that of the drug solution, respectively. In a pharmacokinetic study in rats, the oral administration of Su-SMEDDS formulations prepared with polysorbate 20, Cremophor EL, and polysorbate 80 enhanced the relative bioavailability of the drug by 262%, 458%, and 470%, respectively, compared with the drug suspension. C_max_ increased by 3.65-, 5.86-, and 5.94-fold, respectively. In addition, the authors found an excellent correlation (R^2^ = 0.9679) between in vivo AUC_0–24_ and in situ P_eff_ values. The addition of different P-gp inhibitors resulted in different improvements in the pharmacokinetic properties of Su-SMEDDS formulations. Polysorbate 80 and Cremophor EL were superior to polysorbate 20 in enhancing oral bioavailability and increasing the intestinal permeability of valsartan.

Al-Kandari et al., developed PTX-loaded SMEDDS using Captex 355 (caprylic/capric triglyceride), Cremophor EL, Tween 80, and PEG 400 to improve PTX solubility and membrane permeability [49]. PTX is an effective taxane compound that has been widely used to treat many types of cancer. PTX is a poorly soluble drug and a P-gp substrate with low permeability, which leads to its low oral bioavailability. In pharmacokinetic studies on rabbits, the oral administration of PTX-SMEDDS resulted in a 4.55-fold increase in bioavailability compared with PTX suspension. This improvement was attributed to the enhanced solubility and dissolution rate of PTX. In addition, the intestinal permeability of PTX was increased due to the inhibitory effect of Cremophor EL and Tween 80 on P-gp. Cyclosporine A, a P-gp inhibitor, was administered orally before the administration of PTX-SMEDDS to create a dual effect. Pretreatment with two doses of cyclosporine A (100 mg × 2) increased PTX bioavailability by approximately 1.7-fold compared with PTX-SMEDDS alone, indicating the combined effect of a SMEDDS formulation (containing excipients as P-gp inhibitors) and pretreatment with a P-gp inhibitor. Previously, PTX-SMEDDS has been developed in many studies and showed similar results [156,157,158]. In another study, PTX- and PTX-curcumin-loaded SNEDDS were prepared using sesame oil, Labrasol, and sodium deoxycholate [159]. Pharmacokinetic studies in rabbits showed an increase of 2.8- and 6.3-fold in the oral bioavailability of PTX- and PTX-curcumin-loaded SNEDDS, respectively, compared with the PTX suspension, whereas the C_max_ was also increased by 4.0- and 6.5-fold, respectively. 

### 4.2. Liposomes

Liposomes are spherical vesicles composed of bilayer membranes, such as those formed by phospholipids (Figure 2a). They can incorporate hydrophilic drugs into the water-soluble central compartment and hydrophobic drugs into the bilayer membrane [160]. Liposomes can enhance oral absorption of hydrophobic drugs through several mechanisms that include mucoadhesion, translocation across mucus layers, improved permeation across the enteric epithelia, endocytosis, uptake by M cells, and lymphatic absorption via chylomicron uptake [161]. In addition, phospholipids and other components in liposomes can inhibit P-gp to further improve the permeability of P-gp substrates. However, liposomes are unstable in the harsh gastrointestinal environment because phospholipids aggregate at low pH and with the presence of enzymes, such as pancreatic lipase. Therefore, liposomes are modified by PEGylation, mucin coating, and polymer coating [135]. Some P-gp substrates have been encapsulated in liposomes to improve drug absorption and oral bioavailability.

In one study, docetaxel was loaded into nanoliposomes prepared from phosphatidylcholine and subsequently coated with folate-grafted and thiolated chitosan to improve the oral absorption and targeted pharmacological activity of the drug [162]. In the everted gut sac permeability analysis, the P_app (A-to-B)_ of docetaxel was increased by 5.87-fold when a specific P-gp inhibitor (verapamil) was used. Without verapamil, the nanoliposomes and thiomer enveloped nanoliposomes increased P_app (A-to-B)_ by 9.62- and 13.12-fold, respectively. The ER also decreased from 5.78 for PTX solution to 2.36 for nanoliposomes and 1.0 for thiomer enveloped nanoliposomes. In rabbits, nanoliposomes and thiomer-enveloped nanoliposomes enhanced oral bioavailability by 6.2- and 13.6-fold, respectively, C_max_ by 4.2- and 10.3-fold, respectively, and t_1/2_ from 33 h to 72 h and 86 h, respectively, compared with the drug suspension. The improvement was due to the increased mucoadhesion, P-gp inhibition by thiolated chitosan, and increased paracellular transport attributed to thiolated chitosan and oleic acid.

Li et al., prepared micelle-in-liposomes containing PTX [90]. Pluronic F127-polyethylenimine (PF127-PEI) copolymer was synthesized prior to the fabrication of PF127-PEI/sodium cholate-based polyion micelles. The PTX-loaded micelles were subsequently encapsulated in the aqueous core of phospholipid-based liposomes. SPIP study using rat intestine revealed 12.8-fold higher P_eff_ of PTX-loaded hybrid liposomes than that of PTX solution. The co-administration of verapamil did not alter P_eff_, indicating that the hybrid liposomes could effectively protect PTX from P-gp efflux. In addition, Pluronic F127 inhibited P-gp efflux and enhanced drug absorption by decreasing ATP levels, reducing ATPase activity, and increasing membrane fluidity. In a pharmacokinetic study in rats, PTX-loaded hybrid liposomes showed a 4.33-fold increase in oral bioavailability and approximately 3.3-fold increase in C_max_ compared with the PTX solution. 

Similarly, Romana et al., developed a liposome-micelle-hybrid (LMH) carrier system containing lovastatin, a P-gp substrate with poor aqueous solubility and limited intestinal absorption [55]. TPGS micelles (11 nm in diameter) were prepared and subsequently encapsulated in the aqueous core of liposomes. In Caco-2 cell monolayers, LMH increased lovastatin absorption by 1.14-fold, transportation by 2-fold, and cellular uptake by 2.88-fold compared to the free drug due to P-gp inhibition. However, no pharmacokinetic study was conducted to evaluate the effects of LMH.

Table 2 summarizes the previous features of some emulsions, SMEDDS, SNEDDS, and liposomes with P-gp inhibitory effects to enhance the oral absorption and bioavailability of various P-gp substrates.

### 4.3. SLNs and NLCs

SLNs and NLCs are lipid-based nanoparticles with a solid matrix, which have emerged as alternatives to emulsions, liposomes, and polymeric nanoparticles [163]. SLNs are prepared from solid lipids, surfactants, and co-surfactants. In contrast, in NLCs, liquid oils are added to create imperfect structures that can accommodate more drug (Figure 2a) [164]. NLCs can increase drug loadings and reduce drug expulsion during storage compared to SLNs [163]. SLNs and NLCs are produced from physiological and biodegradable lipids and other generally recognized as safe (GRAS) materials. Therefore, they are both safe and biocompatible. They can also increase the solubility and stability of drugs encapsulated in solid matrices. SLNs and NLCs can be prepared by various methods that include high-pressure homogenization, emulsion/solvent evaporation, microemulsion, phase inversion, and solvent injection [165,166]. SLNs and NLCs can enhance drug absorption and the bioavailability of hydrophilic drugs through several mechanisms, such as uptake by M cells [135], lymphatic absorption from enterocytes via chylomicron uptake [148,149], paracellular transport through tight junction opening [142], and receptor-mediated endocytosis and transcytosis of enterocytes [143,167]. In the case of P-gp substrates, lipids and surfactants in SLNs and NLCs could also inhibit P-gp efflux and increase drug permeation [168]. SLNs and NLCs have been widely used for the encapsulation of various drugs, and many of them are P-gp substrates [169]. 

Another P-gp substrate is andrographolide, which is a diterpenoid from the leaves and roots of *Andrographis paniculate*, which has inhibitory effects on inflammation, cancer, and hyperlipidemia. Low water solubility and P-gp efflux results in poor bioavailability and limits its pharmacological function. Andrographolide-SLNs were prepared to enhance drug absorption and bioavailability [170]. Compritol ATO888, lecithin, glyceryl monostearate, and Tween 80 were used to produce SLNs. In Caco-2 cell monolayers, P_app (A-to-B)_ of andrographolide-loaded SLNs was approximately 2-fold higher than that of andrographolide, whereas ER was reduced by 4.13-fold (from 5.7 to 1.38), indicating the enhanced permeability of drug-loaded SLNs. In addition, the P_app_ value of andrographolide at 37 °C was 2.03-fold higher than that at 4 °C, indicating that drug transport was mediated by the carrier and energy. On the other hand, the P_app_ values of SLNs at 37 °C and 4 °C were similar, confirming passive transport or endocytosis of SLNs. The addition of the specific P-gp inhibitor verapamil increased the P_app (A-to-B)_ and decreased the P_app (B-to-A)_ of the andrographolide solution, but it did not affect the values of drug-loaded SLNs. Thus, the encapsulation of andrographolide in SLNs could protect the drug from P-gp efflux. In addition, the bioavailability and C_max_ of andrographolide-loaded SLNs were 2.41- and 3.63-fold higher, respectively, than those of the drug suspension. In addition to endocytosis, M cell uptake, and lymphatic absorption via chylomicron uptake due to the fabrication of the drug into SLNs, drug absorption can be improved by Tween 80, which is a coating layer of SLNs that inhibits P-gp.

In another study, Ji et al., developed curcumin-loaded SLNs with P-gp inhibitor excipients (TPGS and Brij 78) to enhance curcumin solubility, absorption, and bioavailability using an emulsification and low-temperature solidification method [171]. Curcumin is an active component extracted from turmeric rhizomes that possesses anti-inflammatory, antioxidant, antitumor, and antimicrobial activities. The pharmacokinetic properties of curcumin are less favorable due to its poor aqueous solubility, low gastrointestinal absorption, and rapid metabolism. The curcumin-loaded SLNs were optimized by the Plackett–Burman screening design and Box–Behnken experiment design. In an SPIP study, the P_eff_ value of curcumin for SLNs was 1.64-fold higher than that of curcumin solution. Curcumin-Brij 78 solution and curcumin-Brij 78-TPGS solution could also increase the P_eff_ values of curcumin by 1.28- and 1.39-fold, respectively, indicating P-gp inhibition by Brij 78 and TPGS. In vivo pharmacokinetic studies in rats revealed increases in oral bioavailability (9.42-fold), C_max_ (3.54-fold), time to reach C_max_ (T_max_, from 0.58 h to 5.71 h), and MRT (3.59-fold) of curcumin-loaded SLNs compared with curcumin suspension. In this study, the sustained release of curcumin-loaded SLNs prolonged the contact time of the drug in the intestine, whereas the nanosized particles could increase the drug uptake by enterocytes and M cells. In addition, surfactants have been extensively used to increase drug absorption from the intestine due to their enhanced drug solubility and membrane fluidity. The surfactants Brij 78 and TPGS might disrupt tight junctions and facilitate paracellular transport. Notably, they inhibited P-gp efflux, possibly by affecting the P-gp binding sites of curcumin, inhibiting P-gp ATPase or reducing P-gp expression.

Lumefantrine is an antimalarial drug that induces parasite death by inhibiting heme detoxification. It is a substrate of P-gp, which partly results in its poor and variable oral bioavailability (4–11%) and limits its activity [172]. The optimized formulation containing stearic acid, caprylic acid, TPGS, and poloxamer 188 showed an increase in the effective permeability coefficient (P_eff_) by 3-fold in an SPIP study compared with that of the drug solution [173]. The absorption rate constant (K_a_) of lumefantrine was also increased by 2.96-fold. In mice, the optimized drug-loaded SLNs increase in C_max_ by 2.7-fold and bioavailability by 2.2-fold compared to the drug suspension. The higher intestinal permeability and enhanced oral bioavailability of lumefantrine were due to the presence of the non-ionic surfactants poloxamer 188 and TPGS, which have a P-gp inhibitory effect. In addition, the particle size of SLNs was approximately 350 nm, which resulted in increases in the specific surface area, drug saturation solubility, and dissolution rate. In addition, endocytosis by enterocytes, M cell uptake, and lymphatic absorption of SLNs were also involved in the enhancement of lumefantrine oral bioavailability.

Khurana et al., developed a hybrid system of phospholipid complex (PLCs) loaded in NLCs for the encapsulation of mangiferin, which is an antioxidant extracted from the leaves and stem bark of Mango [86]. It has low and variable oral bioavailability due to poor aqueous solubility, high P-gp efflux, inconsistent gastrointestinal absorption, and high hepatic first-pass metabolism. PLCs were prepared using Phospholipon 90 G (soybean lecithin in 90% phosphatidylcholine) by the solvent evaporation method. Then, NLCs were produced using hot emulsification and ultrasonication with Compritol 888 ATO as a solid lipid, Labrafil M 2125 as oil, Tween 80 as a surfactant, and soy lecithin as a cosurfactant. In Caco-2 cell monolayers, PLCs and PLCs-NLCs showed 9.4- and 10.1-fold increases in P_app_, respectively, compared with the plain mangiferin solution after 3 h incubation, which was attributed to the P-gp inhibitory effects of phospholipids and surfactants (Tween 80 and lecithin). An in situ SPIP study revealed increases in the P_eff_ values of PLCs and PLCs-NLCs compared with the drug solution and drug + verapamil solution. In vivo pharmacokinetic studies revealed considerable increases in C_max_ (4.8-fold) and bioavailability (2.1-fold) of PLCs-NLCs compared with the drug solution. The PLCs-NLCs did not show much enhancement over PLCs, with only a 1.2-fold increase in bioavailability. However, the PLCs-NLCs were superior to PLCs in Caco-2 cells as determined by P-gp efflux assay using the Chemicon MDR dye efflux assay kit (2.1-fold). Therefore, the hybrid system could enhance oral bioavailability by inhibiting P-gp efflux and other mechanisms.

Aripiprazole is an atypical antipsychotic used for the treatment of schizophrenia associated with negative symptoms. It has poor water solubility and undergoes P-gp efflux and extensive hepatic metabolism, which reduces its efficacy and increases dose-related side effects. Aripiprazole-loaded SLNs were prepared using tristearin, sodium taurocholate, and Tween 80 to improve drug absorption and oral bioavailability [50]. The oral administration of aripiprazole-loaded SLNs to rats increased bioavailability by 1.6-fold and C_max_ by 2-fold compared with the aripiprazole suspension. Although no permeability studies were carried out, these increases might be due to the increase in drug absorption via lymphatic transport and uptake by M cells. In addition, Tween 80 in the SLN composition could inhibit P-gp efflux, resulting in enhanced drug absorption. 

Darunavir is a protease inhibitor used for the treatment of HIV. The drug has low oral bioavailability due to its lipophilic nature, P-gp efflux, and metabolism by cytochrome P450 enzymes. It was incorporated into SLNs using the high-pressure homogenization method with hydrogenated castor oil as a lipid and sodium oleate as a surfactant [91]. The darunavir-loaded SLNs were further coated with a peptide that could increase the binding and cellular internalization of SLNs to CD4+ cells. Caco-2 cellular uptake of SLNs was reduced in the presence of chlorpromazine and nystatin, but it was unchanged in the presence of amiloride, indicating that the uptake of SLNs was by clathrin- and caveolae-mediated endocytosis and not by micropinocytosis. The P_app_ of darunavir in Caco-2 cell monolayers increased by 4.04-fold after incorporation into SLNs, which was ascribed to endocytosis. Similarly, an in situ SPIP study using rat stomach and duodenum revealed increases in the AUC by 2.50- and 2.51-fold, respectively. After oral administration to rats, peptide-coated and uncoated darunavir-loaded SLNs showed increases in C_max_ (2.61–2.69-fold) and bioavailability (4.81–5.69-fold) compared with the drug suspension. In the biodistribution study, peptide-coated SLNs exhibited 1.5- to 3.5-fold higher accumulation of darunavir in the brain compared with the drug suspension, which could be attributed to the inherent ability of SLNs to cross the BBB via the tight junction opening and absorption through capillary walls via receptor-mediated endocytosis. In particular, the darunavir amount in Peyer’s region was 4.12-fold higher than that in non-Peyer’s region, suggesting the efficient uptake of SLNs via Peyer’s patch, leading to higher lymphatic absorption by the mesenteric lymph nodes and spleen. Thus, the improved bioavailability of darunavir was ascribed to the various effects of SLNs. 

Tilmicosin is a macrolide antibiotic that has been widely used in veterinary clinics to treat bacterial infections due to its low inhibitory concentration and high distribution volume. Its oral bioavailability often varies in different animals as a result of incomplete oral absorption. Zhou et al., recently developed tilmicosin-loaded SLNs using a hot melt with an ultrasonic emulsification method [89]. Carnauba wax was used as a solid lipid, whereas polyvinyl alcohol was a surfactant in the optimized SLNs. In an SPIP study using rat intestines, SLNs increased the P_app_ (2.08-fold) and K_a_ (1.67-fold) of tilmicosin in the duodenum, whereas those in the jejunum and ileum were unchanged. The addition of 1% Tween 80 improved the P_app_ and K_a_ of tilmicosin-loaded SLNs by 2.32-and 2.13-fold, respectively, which indicated the inhibition of P-gp by Tween 80. The optimum tilmicosin-loaded SLNs were further loaded into enteric granules containing sucrose, starch, starch paste, and Tween 80. The granules were coated with polyacryl resin II to control drug release, as the polymer was dissolved at pH >6. The in vitro release study revealed that the enteric granules effectively delivered more tilmicosin-loaded SLNs to the duodenum. Pharmacokinetic study in pigs showed that enteric granules containing SLNs increased oral bioavailability (2.72-fold) and C_max_ (2.92-fold) compared with a commercial premix. This was due to the combination of duodenum-targeted release by the enteric coating, P-gp inhibition of Tween 80, and other absorption mechanisms resulted from the SLNs.

**Figure 3:**
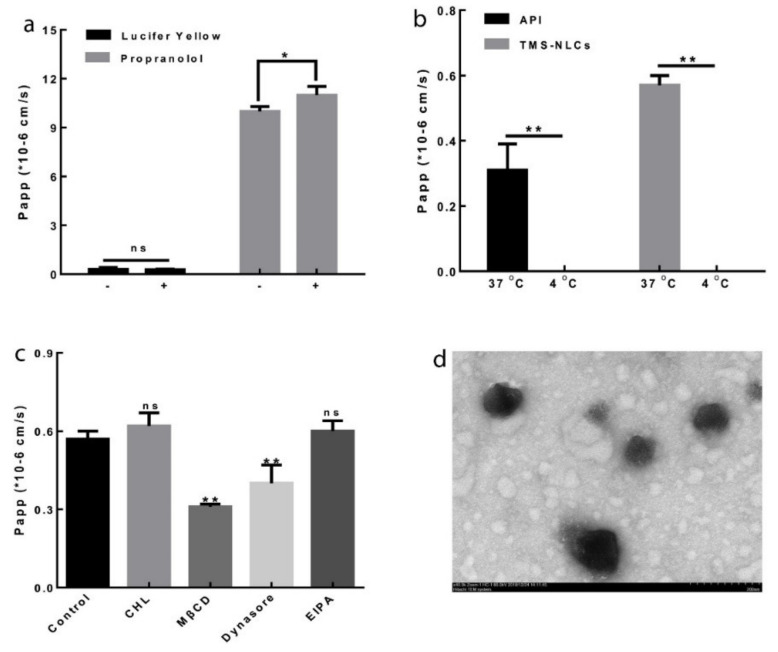
Pathway of tilmicosin-loaded NLCs transport into Caco-2 cell monolayers. (**a**) Effect of NLCs on P_app_ of Lucifer Yellow and propranolol (+ and -: treated with and without tilmicosin-loaded NLCs, respectively). (**b**) The P_app_ of tilmicosin-loaded NLCs (TMS-NLCs) and tilmicosin (API) at 4 °C and 37 °C. (**c**) The effect of various endocytosis inhibitors on the P_app_ of TMS-NLCs. CHL: chlorpromazine, MβCD: methyl-β-cyclodextrin, EIPA: 5-(N-ethyl-N-isopropyl)-amiloride, ns: no significance. * *p* < 0.05 and ** *p* < 0.01 compared with control. (**d**) After 4 h of transcellular transport, TMS-NLCs from the A-to-B side transport fluids were collected to observe the nanoparticle morphology by TEM. Scale bar was 200 nm. Reprinted from [174], Copyright (2019), with permission from Elsevier.

In another study, tilmicosin-loaded NLCs were prepared using high shear and ultrasonic methods with stearic acid as a solid lipid, oleic acid as oil, and Tween 80 as a surfactant [174]. Pharmacokinetic analysis in piglets showed a slight increase (1.42-fold) in the oral bioavailability of tilmicosin-loaded NLCs compared with the drug suspension. In Caco-2 cell monolayers, NLCs increased the P_app (A-to-B)_ of tilmicosin by 1.84-fold and slightly reduced P_app (B-to-A)_ by 1.14-fold. The ER also decreased from 3.04 to 1.45 when using NLCs. The addition of verapamil enhanced P_app (A-to-B)_ and reduced the ER of the drug solution. However, it did not affect the values of tilmicosin-loaded NLCs, indicating that NLCs escaped P-gp efflux. The P_app_ value of Lucifer Yellow, a paracellular marker, was unchanged when co-incubated with NLCs, whereas that of propranolol, a transcellular transport marker, increased after co-incubation (Figure 3a). These results suggested that the transport of tilmicosin-loaded NLCs occurred mainly through the transcellular pathway rather than the paracellular pathway. The P_app_ values of tilmicosin and tilmicosin-loaded NLCs at 4 °C were lower than those at 37 °C (Figure 3b). Since at 4 °C, cell metabolism is inhibited and pinocytic/endocytic uptake is inactivated, the data suggested that NLCs mainly entered cells by an energy-dependent active transport route. The authors also identified that tilmicosin-loaded NLCs were mainly internalized by Caco-2 cells through the caveolae/lipid raft-mediated endocytosis pathway (Figure 3c). Transmission electron microscopy revealed the presence of intact NLCs on the basolateral side (Figure 3d), indicating that they could escape lysosomal degradation. The collective findings indicate that NLCs could improve the oral bioavailability of tilmicosin by enhancing drug solubility and permeability and by decreasing the P-gp efflux.

Sahito et al., also fabricated three types of tilmicosin-loaded NLCs to enhance the oral bioavailability of the drug [175]. NLCs were prepared by high shear and ultrasonic methods with palmitic acid, lauric acid, or stearic acid as a solid lipid, oleic acid as oil, and Tween 20 as a surfactant. In Caco-2 cell monolayers, three NLCs increased the P_app (A-to-B)_ of tilmicosin by 1.69–2.03-fold and reduced ER from 2.29 to 1.561.88. Pharmacokinetic study in chickens revealed increases in bioavailability (2.04-fold) and C_max_ (1.36-fold) of tilmicosin-loaded stearic acid-NLCs compared with the drug suspension. NLCs prepared with palmitic acid and lauric acid exhibited only slight improvement. However, the inhibition of P-gp efflux by stearic acid-NLCs was not the strongest among the three formulations. We hypothesized that the three NLCs with different solid lipid components might have different effects on the uptake of NLCs through M cells, enterocyte endocytosis, and the intestinal lymphatic system via the chylomicron uptake mechanism.

Linagliptin is a dipeptidyl peptidase-4 (DPP-4) inhibitor. It is a novel anti-diabetic drug with low oral bioavailability due to first-pass metabolism and P-gp efflux. To increase its oral bioavailability, poloxamer-188 and Tween 80 were used as surfactants and P-gp inhibitors for the preparation of linagliptin-loaded SLNs [43]. SLNs were prepared by hot homogenization and ultrasonication. Palmitic acid, poloxamer 188, and Tween 80 were used as the lipid, surfactant, and cosurfactant, respectively. In Caco-2 permeability studies, the permeability of linagliptin-loaded SLNs was 1.74-fold higher than that of the linagliptin solution. Similarly, in SPIP and rat everted gut sac permeability studies, the increases in permeability were 1.82- and 1.76-fold, respectively. In rats, the pharmacokinetic properties of linagliptin-loaded SLNs were improved, including the extended T_max_ (12 h versus 4 h), higher C_max_ (2.4-fold), longer MRT, and enhanced relative bioavailability (3-fold). Pharmacodynamic studies in rats revealed significantly lower blood glucose levels in linagliptin-loaded SLNs at all times. Thus, linagliptin-loaded SLNs showed an increased and prolonged pharmacological effect because the SLNs avoided first-pass metabolism by lymphatic transport. In addition, P-gp inhibitors increased drug absorption and oral bioavailability. The improved absorption reduced the administration dose, dose-related side effects, and administration frequency. 

**Figure 4:**
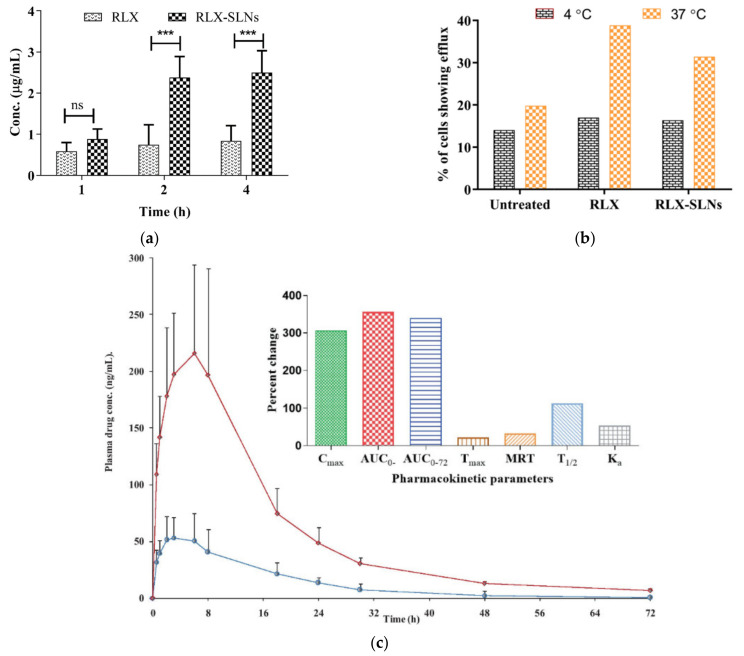
Effects of raloxifene (RLX)-loaded SLNs on (**a**) Caco-2 cellular uptake, (**b**) P-gp efflux using the Chemicon MDR dye efflux assay kit, and (**c**) pharmacokinetic properties of RLX (red curve: RLX-loaded SLNs and blue curve: RLX suspension). *** *p* < 0.001, ns, not significant. Reprinted by permission from Springer Nature, Drug Delivery and Translational Research [87], Copyright © 2021.

Recently, Jain et al., developed SLNs loaded with raloxifene, an estrogen receptor modulator that is approved for breast cancer management [87]. This drug shows poor and inconsistent oral bioavailability in humans due to its low aqueous solubility, P-gp efflux, pre-systemic glucuronide conjugation, and extensive first-pass metabolism. SLNs were prepared by the solvent diffusion method with glyceryl monostearate and Compritol 888 ATO as lipids, TPGS-1000 as a surfactant, and Phospholipid S-100 as a cosurfactant. In Caco-2 cells, the cellular uptake of raloxifene was increased by 3.18-fold after encapsulation in SLNs (Figure 4a). In the P-gp efflux study using the Chemicon MDR dye efflux assay kit, the percentage of cells showing efflux was reduced when incubated with raloxifene-loaded SLNs (Figure 4b). This was ascribed to the inhibitory effect of TPGS-1000 on P-gp activity. Pharmacokinetic study in rats indicated the improved biopharmaceutical performance of raloxifene-loaded SLNs compared with that of the pure drug (Figure 4c), with increases in C_max_ (4.06-fold), bioavailability (4.56-fold), and t_1/2_ (2.12-fold). Thus, SLNs improved permeability and reduced P-gp efflux, resulting in enhanced oral absorption and bioavailability of raloxifene.

Recently, Qin et al., developed PLHNs, which combined the advantages of SLNs and polymeric NPs in the drug delivery to enhance the oral absorption of PTX [126]. PTX-loaded PLHNs prepared by a self-assembly method were composed of a glyceryl monooleate lipid core and a polymeric shell of Soluplus, which is a polyvinyl caprolactam-polyvinyl acetate-PEG graft copolymer. Chitosan was used to decorate the PLHNs for mucoadhesion and stability enhancement, whereas a P-gp inhibitor (TPGS, Pluronic P123, or Solutol HS15) was included to further increase drug absorption. Among the three P-gp inhibitors, TPGS-incorporated PLHNs possessed the strongest absorption enhancing effect, with 2.46- (in the duodenum) and 1.35-fold (in the ileum) increases in P_app_ values compared with that of Taxol. TPGS, Pluronic P123, and Solutol HS15-incorporated PLHNs enhanced the oral bioavailability of PTX in rats by 7.51-, 6.91-, and 7.06-fold, respectively, compared to Taxol. In addition, T_max_ and half-life were extended for all the PLHN formulations. Several factors could contribute to the prominent enhancement of PTX absorption, such as the increased solubility of PTX, improved uptake of PTX via Peyer’s patches into the blood through lymphatic circulation due to the presence of glyceryl monooleate, prolonged retention time in the gut due to the bioadhesion of chitosan, and P-gp mediated inhibition of TPGS, Pluronic P123, and Solutol HS15.

Table 3 summarizes the components and major results of SLN and NLC formulations with P-gp inhibitory effects on the enhancement of oral absorption and bioavailability of various P-gp substrates.

### 4.4. Micelles

Micelles are spherical amphiphilic structures formed by the self-assembly of amphiphilic molecules (Figure 2a). They have a hydrophobic core that is suitable for the encapsulation of poorly water-soluble drugs. A hydrophilic shell can stabilize the hydrophobic core and make the micelles water-soluble [176]. Micelles are nanosized colloidal dispersions with a diameter of 10–200 nm, which avoids elimination by the reticuloendothelial system and increases their circulation time [177]. Polymeric micelles are produced by increasing the polymer concentration above the corresponding CMC. Micelles have the advantages of easy drug encapsulation and easy surface manipulation. Drugs can be encapsulated by chemical covalent attachments or physical methods. In chemical methods, the drugs are covalently cross-linked with polymers, which considerably improves circulation kinetics, biodistribution, and the accumulation of micelles at target sites. However, cross-linking chemical reactions can be challenging and complicated. However, physical methods are practical and simpler. Several widely used techniques include solvent evaporation, oil-in-water emulsion, direct dissolution, dialysis, and freeze-drying [178]. Many surfactants with P-gp inhibitory activity can be used for the preparation of micelles. These include Tween 80, TPGS, Cremophor EL, and Pluronic 85. They can effectively improve the intestinal permeability of P-gp substrates [133]. In addition, the encapsulation of hydrophobic drugs in micelles increases their aqueous solubility and affinity to the intestinal membrane [28]. Micelles are also absorbed into systemic circulation through the lymph transport pathway [44]. Nanosized structures can increase the active uptake of drugs across intestinal epithelial cells by enterocyte transcytosis or endocytosis [134]. Micelles with neutral and positive charges have a higher affinity for intestinal epithelia than those with negative charges, which may contribute to the improvement of transport across the intestinal epithelia. The drug residence time in systematic circulation can also be prolonged [127]. 

Some P-gp substrates have been formulated into micelles to improve their solubility, absorption, and bioavailability. For example, PTX is one of the most studied P-gp substrates. Zhang et al., developed three different PTX-loaded micelles using a one-step self-assembly method: (i) PTX micelles containing PTX and Solutol HS15, (ii) antiresistant PTX micelles containing PTX, TPGS, and Solutol HS15 (ratio of 1:30:60), and (iii) super-antiresistant PTX micelles containing PTX, bromotetrandrine (W198), TPGS, and Solutol HS15 (ratio of 1:1:30:60) [133]. W198 is a P-gp inhibitor with low specificity and toxicity. It can increase the permeability of PTX across the intestinal barrier and enhance active uptake [145]. In the Caco-2 cell monolayer permeation study, the permeability of PTX micelles, antiresistant PTX micelles, and super-antiresistant PTX micelles were 2.52-, 1.92-, and 2.45-fold higher, respectively, than that of Taxol. The authors also investigated the permeability mechanism of super-antiresistant PTX micelles across Caco-2 cell monolayers. The results showed that caveolae-mediated endocytosis, micropinocytosis, and cholesterol-dependent endocytosis were the main uptake pathways of Taxol and super-antiresistant PTX micelles. Another transport pathway for super-antiresistant PTX micelles was clathrin-mediated endocytosis. Pharmacokinetic study in rats revealed an almost 2-fold increase in the AUC and C_max_ of the highly resistant PTX micelles compared with those of the Taxol group following oral administration. This enhancement was possibly due to the presence of TPGS and W198 in the micelles, which inhibited P-gp efflux and intestinal metabolism. The in vivo antitumor efficacy study was evaluated using MCF-7/Adr xenograft mouse models. The tumor inhibition rate of super-antiresistant PTX micelles was 45.90 ± 10.47% and 44.62 ± 11.15% for intravenous and oral administration routes, respectively, which were higher than the rate of Taxol. The improved antitumor efficacy of orally administered super-antiresistant PTX micelles was due to the P-gp inhibitory activity of TPGS and W198, increased permeability across the intestinal barriers, and enhanced oral bioavailability. In addition, the small size (approximately 13 nm) of the super-antiresistant PTX micelles increased their penetration into tumor tissues. 

Similarly, Zhao et al., recently developed W198/PTX multistage oral micelles by ultrasonication using Tween 80 and Cremophor EL as surfactants [28]. Compared with PTX micelles alone, W198/PTX micelles exhibited 10-fold higher cellular uptake in Caco-2 cells and approximately 5.7-fold higher oral bioavailability in rats. The C_max_ of PTX in the W198/PTX micelle group was also 2.80-fold higher than that in the PTX micelle group. The surfactants Tween 80 and Cremophor EL in micelles could improve the water solubility of PTX and increase its affinity with the intestinal membrane. They are also P-gp inhibitors that can decrease P-gp activity. Co-administration of the P-gp inhibitor W198 further reduced P-gp efflux by decreasing ATPase, resulting in the improvement of intestinal absorption and permeability. 

Other groups have also developed micelles loaded with PTX. Sze et al., used PEG-block-poly(ε-caprolactone) (PEG-b-PCL) to prepare PTX and Rhodamine 123-loaded polymeric micelles [82]. Micelles prepared with PEG17-b-PCL5 (low MW) showed increased cellular uptake of the P-gp substrates PTX and Rhodamine 123, and the non-P-gp substrate coumarin compared with PEG114-b-PCL88 (high MW) in MDCKII and MDCK-MDR1 cell lines. In the MDCK-MDR1 cell monolayers, the apparent permeability coefficient of Rhodamine 123 in the PEG17-b-PCL5 micelles was 1.62-fold higher than that in the Rhodamine 123 solution. Pharmacokinetic study in rats showed that at a dose of 10 mg/kg, the C_max_ and oral bioavailability of PTX in PEG17-b-PCL5 micelles increased by 3.61- and 2.61-fold, respectively, compared with those in PEG114-b-PCL88 micelles. However, there was no control group with PTX suspension or Taxol to evaluate the enhancement of pharmacokinetic properties by the micelles.

N-octyl-N’-phthalyl-O-phosphoryl chitosan (OPPC), an amphipathic chitosan derivative, was used to prepare micelles loaded with PTX [179]. The micelles enhanced the stability and integrity of the mouse gastrointestinal tract in an in vivo fluorescence imaging study. In addition, the micelles showed effective transport and accumulation into Caco-2 cells via P-gp inhibition mediated by OPPC as well as clathrin/caveolae-mediated endocytosis. The P-gp inhibitory effect of OPPC was attributed to a decrease in P-gp ATPase activity and a reduction in membrane fluidity. PTX-loaded OPPC micelle capsules showed an absolute bioavailability of 31.45 ± 7.73%, which was 5.5-, 3.8-, and 1.3-fold higher than Taxol, Taxol + verapamil, and PTX-loaded OPPC micelles via the duodenum, respectively. PTX-loaded OPPC micelles increased the solubility of PTX and its stability in vivo. In addition, PTX absorption was enhanced by the OPPC micelles and the possible P-gp inhibitory effect of the OPPC polymer. 

Wang et al., used a carboxymethyl chitosan-rhein conjugate to prepare polymeric micelles of PTX using ultrasonication and dialysis methods [134]. PTX-loaded micelles could enhance the absorption of PTX in the intestine compared with Taxol, Taxol + verapamil, and Taxol + carboxymethyl chitosan-rhein conjugate in SPIP studies. Notably, the micelles did not cause significant intestinal villi injury compared with verapamil due to their endocytosis transport route. Thus, micelles can bypass P-gp efflux and cross the intestinal epithelial cells. In Caco-2 cell uptake assays, the micelles were taken up into the enterocytes as a whole without being affected by P-gp. In addition, fluorescence-labeled micelles were absorbed into the intestinal villi during the local biodistribution evaluation. 

In a recent study, a gallic acid-chitosan-TPGS (GA-CS-TPGS) copolymer was synthesized for the preparation of micelles loaded with PTX [137]. This copolymer has multiple functions associated with its components. Chitosan has strong mucoadhesive properties, and it has an inhibitory effect on liver enzyme CYP3A, whereas TPGS can inhibit P-gp efflux. The PTX-loaded micelles showed increased permeability in the Caco-2 cell monolayer model compared with the PTX suspension. The micelles were bioadhesive due to the cationic profile of the GA-CS-TPGS copolymer and strongly inhibited CYP3A enzyme activity, which resulted in reduced first-pass metabolism. The oral bioavailability and C_max_ of PTX were improved by approximately 3.80- and 3.95-fold, respectively, after PTX encapsulation in micelles. In addition, the PTX-loaded micelles enhanced the efficacy against lung tumors compared to Taxol in mice. This improvement was attributed to the multiple functions of the GA-CS-TPGS copolymer as well as the enhanced solubility and permeability of PTX by the micelles.

**Figure 5:**
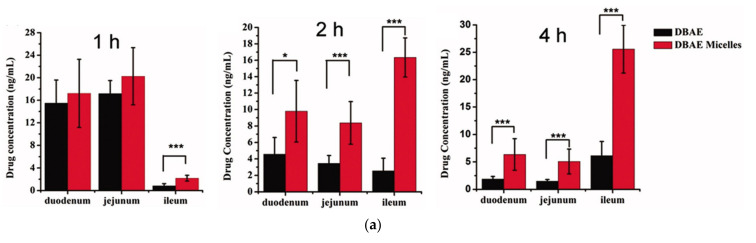

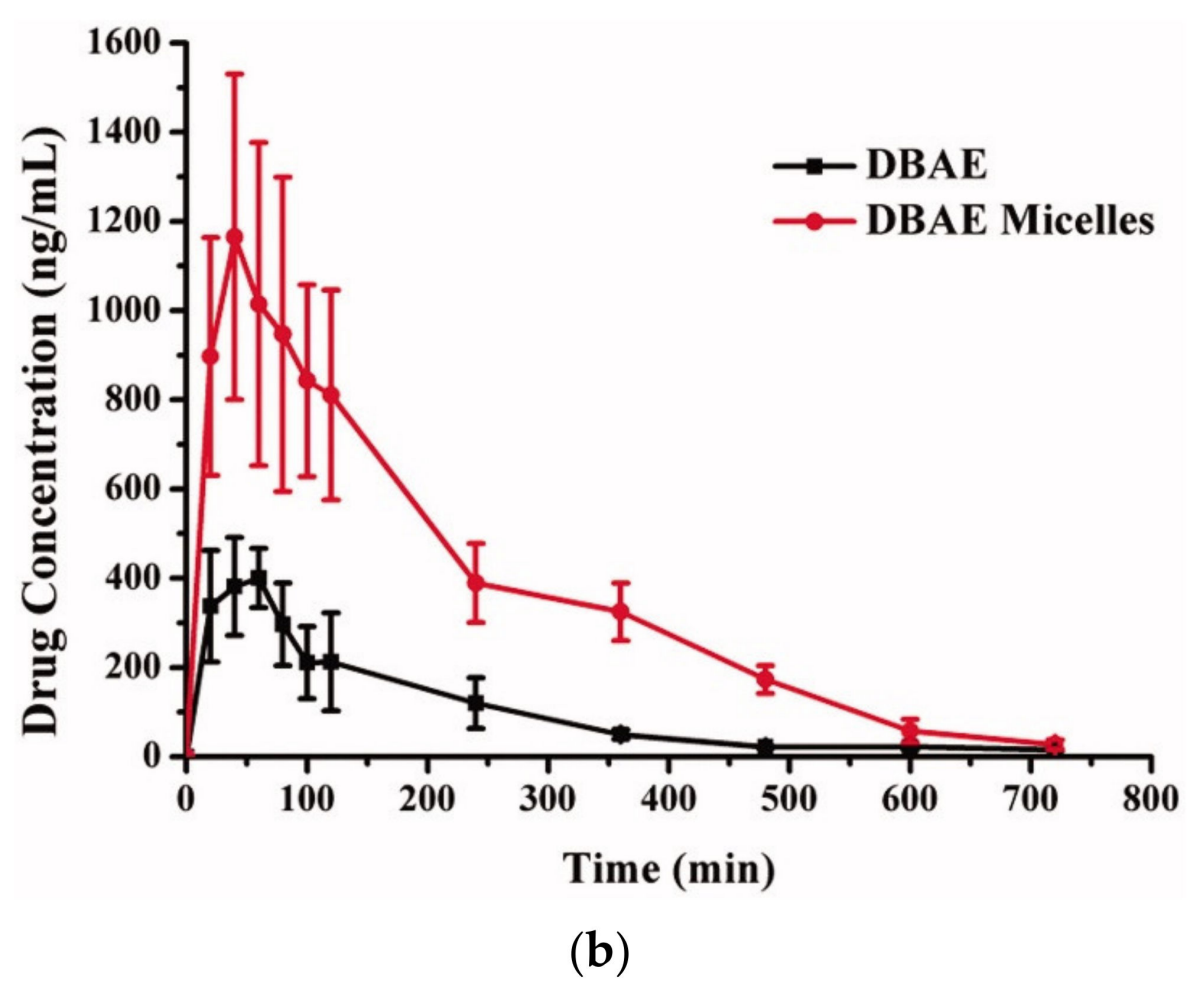
Effects of mixed micelles on (**a**) intestinal absorption and (**b**) pharmacokinetic properties of DBAE. * *p*  <  0.05, *** *p*  <  0.01. Reprinted from [127] by permission of Taylor &Francis Ltd. (London, UK).

In addition to PTX, other P-gp substrates have also been encapsulated into micelles to enhance oral bioavailability. Dabigatran etexilate (DBAE) is an anticoagulant. Advantages of DBAE include oral administration, minimal interactions with other drugs and foods, and predictable pharmacokinetic and pharmacodynamic profiles. It is a poorly soluble drug with an aqueous solubility of approximately 4.7 μg/mL at 37 °C and is a substrate of P-gp, which leads to poor oral absorption and low oral bioavailability. The drug is administered orally at a daily dose of 300 mg, which can increase the risk of bleeding. A mixed micelle system was prepared from Soluplus and TPGS to enhance the oral absorption and bioavailability of the drug [127]. In Caco-2 cell monolayers, the cellular uptake of DBAE-loaded micelles was increased by 2–2.6-fold compared with that of the DBAE suspension. In addition, the intestinal absorption of DBAE from the optimized micelles in rats was enhanced by 8- and 5-fold in the ileum at 2 h and 4 h, respectively (Figure 5a). The authors used a chylomicron flow blocking approach to evaluate the lymphatic transport of DBAE-loaded micelles and found that the micelles were transported into the systemic circulation by both blood and lymph transport pathways. This finding was similar to the previous observation that TPGS could facilitate the secretion of chylomicrons by intestinal epithelial cells and promotion of the intestinal lymphatic transport of lipophilic drugs [180]. The C_max_ and oral bioavailability of the optimized DBAE-loaded micelles in rats were 2.92- and 3.37-fold higher, respectively, than those of DBAE suspension (Figure 5b). The thrombosis inhibition rate of the optimized micelles in rats was 63.18%, which was approximately 4.24-fold higher than that of the DBAE suspension. The enhanced oral bioavailability and therapeutic efficacy of DBAE-loaded mixed micelles were due to several reasons, such as the increased aqueous solubility of the drug, improved affinity between the micelles and intestinal membrane (due to Soluplus, TPGS, and the neutral surface charge of the micelles), and P-gp inhibition by TPGS, which increased drug permeability [181,182]. In addition, the bioavailability could also be improved due to the sustained release property of the micelles in the intestine, which resulted in prolonged drug residence time in systematic circulation. 

In another study, mixed polymeric micelles loaded with cyclosporine A were prepared using curcumin-carboxymethyl chitosan and a low-molecular weight heparin-all-trans-retinoid acid conjugate [44]. Curcumin is a P-gp inhibitor, whereas carboxymethyl chitosan can enhance intestinal absorption due to its biocompatibility and transient widening of the tight junctions. SPIP studies in rats showed that the micelles increased the intestinal absorption of cyclosporine A compared with the drug suspension and mixture of drug and curcumin–carboxymethyl chitosan in the duodenum and jejunum segments. The micelles encapsulated the drug in the core and protected it from P-gp recognition to reduce P-gp efflux. The micelles also increased the oral bioavailability and C_max_ of cyclosporine A compared with those of cyclosporine A suspension (6.02- and 15.13-fold, respectively). 

Wang et al., synthesized lysine-linked ditocopherol PEG 2000 succinate (PLV_2K_), an amphiphilic copolymer that inhibits P-gp [183]. The polymer was used to prepare micelles loaded with the anticancer drug doxorubicin. In situ SPIP study revealed enhanced K_a_ of doxorubicin by 3.19-, 1.61-, and 1.80-fold in the duodenum, jejunum, and ileum, respectively, after encapsulation in micelles. The micelles improved the membrane permeability of doxorubicin by caveolae-dependent/independent and clathrin-dependent/independent transcytosis in Caco-2 cells. Paracellular transport was not involved, as evidenced by the unchanged TEER. An in vivo pharmacokinetic study in rats revealed 5.6-fold higher bioavailability in the micelles compared to the drug solution.

Kwon et al., employed two P-gp inhibitors, Tween 80 and Pluronic 85, to prepare berberine micelles [45]. Berberine is an isoquinoline alkaloid used for the treatment of bacteria-associated diarrhea and other gastrointestinal infections, which has low bioavailability due to intestinal P-gp efflux. Berberine-loaded Tween 80 and Pluronic 85 mixed micelle formulations (ratio of 1:5:0.5) were prepared using solvent evaporation and the thin-film hydration method. Permeability study in Caco-2 cells revealed a 3.64-fold increased absorption permeability of berberine. The ER decreased significantly (from 7.5% to 1.1% at a berberine concentration of 100 μM) with the introduction of berberine micelles. These results suggest P-gp inhibition of berberine micelle formulation. In a pharmacokinetic study following the oral administration of berberine micelles in rats, the bioavailability of berberine was significantly increased 15.6-fold compared with that of the berberine suspension. These results suggested an increase in berberine absorption when using berberine micelle formulation, which could be attributed to the use of two pharmaceutical excipients with a P-gp inhibitory effect, Tween 80 and Pluronic 85. 

Mixed micelles of Soluplus and TPGS were recently prepared for aripiprazole encapsulation [184]. The parallel artificial membrane permeability assay for the intestine revealed a 2-fold increase in the permeability of aripiprazole compared with that of the free drug. In addition, aripiprazole-loaded mixed micelles increased the oral bioavailability AUC_0–24h_ by 1.60-fold and C_max_ by 1.64-fold of aripiprazole in rats. The findings indicate that TPGS plays a critical role in the inhibition of P-gp, which improves drug permeability, absorption, and bioavailability.

Table 4 summarizes the components and major results of micelles with P-gp inhibitory effects on the enhancement of oral absorption and bioavailability of various P-gp substrates.

### 4.5. Polymeric Nanoparticles (NPs)

Polymeric NPs are nano-colloidal systems produced from different polymers. They have been widely used for the oral delivery of various chemotherapeutic agents. Polymeric NPs have the advantages of high stability in the gastrointestinal tract and the ability to encapsulate a variety of drug molecules [185]. Polymers used for the preparation of polymeric NPs include natural polymers, such as chitosan, gelatin, dextran, albumin, and alginate, and synthetic biodegradable polymers that include polylactic acid, polyglycolic acid, polylactic and polyglycolic acid copolymers (PLGA), polyethylene imine (PEI), polyalkyl cyanoacrylate (PACA), and PCL [186]. Some polymeric NPs, such as chitosan, typically have mucoadhesive properties that increase their residence time and diffusion in the mucus. They are mainly absorbed in the gastrointestinal tract via endocytosis, such as clathrin-dependent endocytosis for various chitosan and PLGA-NPs. Phagocytosis by M cells is also involved in the absorption of polymeric NPs via lymphatic organs [187]. In addition, some components in polymeric NPs can open tight junctions to increase the paracellular transport of drugs, such as chitosan and its derivatives [185]. Many P-gp substrates have been encapsulated into polymeric micelles to bypass P-gp efflux, thereby enhancing oral bioavailability. 

Poly(methyl vinyl ether-co-maleic anhydride) is another polymer that has been used for the oral delivery of PTX [188]. PEGs with different MWs were added to prepare three types of NPs: PTX-NP2, PTX-NP6, and PTX-NP10 (with PEG 2000, PEG 6000, and PEG 10,000, respectively). Uncoated NPs (PTX-NP) were also fabricated. Ex vivo permeability studies using Ussing chambers revealed that the three NPs increased P_app_ by 7.2-, 7.3-, and 3.3-fold, respectively, compared with Taxol. In the absence of PEG, NPs increased P_app_ by 1.8-fold. When Taxol was orally administered to rats, PTX was not detected in the plasma. In PTX-NP lacking PEG, the absolute oral bioavailability was 9.1% compared with intravenous Taxol. With PEG, the absolute oral bioavailability increased to 16%, 40%, and 70% for PTX-NP10, PTX-NP6, and PTX-NP2, respectively. The results were related to the bioadhesive properties of the three PEGylated NPs. In vivo bioadhesion analyses revealed more adherent NPs in the stomach, small intestine, and cecum of rats with the PTX-NP2 and PTX-NP6 preparations compared with PTX-NP10 and PTX-NP. The findings indicated that mucoadhesion by PEG was critical to enhance drug permeability and oral bioavailability. The P-gp inhibitory effect of PEG was also involved in the enhancement.

Zhang et al., synthesized a novel poly(methyl vinyl ether-co-maleic anhydride)-graft-hydroxypropyl-β-cyclodextrin amphiphilic copolymer (CD-PVM/MA), which combines the bioadhesiveness of PVM/MA and the inhibitory effect of CD on P-gp and cytochrome P450 [104]. The copolymer was used to prepare NPs loaded with tacrolimus, an immunosuppressant used to prevent transplant rejection. A biodistribution analysis revealed good bioadhesion to the rat intestinal wall. Clathrin- and caveolae-mediated endocytosis were identified in the cellular uptake of NPs. The authors also performed a mesenteric lymph duct ligation experiment to verify the mesenteric lymphatic transport of the NPs. Approximately 58% of the drug was absorbed via the lymphatic route, indicating its essential role in the oral absorption of NPs. In the pharmacokinetic study in rats, the NPs showed increases in C_max_ (11.54-fold) and bioavailability (9.51-fold) compared with the drug solution at the same dose (10 mg/kg).

PLGA-NPs were developed for the delivery of rapamycin, which is a macrolide obtained from *Streptomyces hygroscopicus*, for breast cancer treatment [189]. The drug is a P-gp substrate with low water solubility and oral bioavailability. Piperine was also co-delivered with rapamycin because of its P-gp inhibitory effect and anticancer activity. An everted gut sac permeability study revealed higher permeability of rapamycin-loaded NPs with and without piperine compared to rapamycin suspension. In addition, the permeability increased 5-fold when piperine was co-loaded in the NPs, which confirmed the P-gp inhibitory effect of piperine. Pharmacokinetic study in rats revealed that the rapamycin-loaded NPs with and without piperine increased bioavailability 3.5- and 4.8-fold, respectively, compared with rapamycin suspension.

Another study reported the encapsulation of rapamycin in TPGS-lecithins-zein NPs [190]. The NPs had a core of drug-loaded zein and a shell of lecithin and TPGS. The coumarin-6-loaded TPGS-lecithins-zein NPs exhibited 2.43- and 1.48-fold higher uptake in Caco-2 cells compared with free coumarin-6 and coumarin-6-loaded lecithins-zein NPs, respectively, indicating the critical role of TPGS in intracellular uptake. In Caco-2 cell monolayers, the encapsulation of rapamycin into TPGS-lecithins-zein NPs and lecithins-zein NPs increased the P_app_ of the drug by 1.39- and 1.32-fold and decreased ER from 1.47 to 1.02 and 1.12, respectively. In situ SPIP study exhibited similar results; K_a_ and P_eff_ in all segments of rat intestine were in the order of rapamycin-loaded TPGS-lecithins-zein NPs > rapamycin-loaded lecithins-zein NPs > rapamycin solution. Pharmacokinetic study revealed that rapamycin-loaded TPGS-lecithins-zein NPs and rapamycin-loaded lecithins-zein NPs showed increases in C_max_ (2.04- and 1.70-fold, respectively) and bioavailability (2.64- and 2.12-fold, respectively) compared with the drug solution. In this study, the structure of NPs might have mimicked lipoprotein, which promotes cellular uptake and oral absorption in rats, whereas TPGS as a P-gp inhibitor could reduce drug efflux.

Tarig et al., also prepared PLGA-NPs for the oral delivery of epirubicin, a P-gp substrate anthracycline analog with poor oral bioavailability [191]. The NPs were superficially decorated with PEG (EPI-PNPs) and mannosamine (EPI-MNPs). The authors used confocal laser scanning microscopy and flow cytometry analyses to reveal the enhanced drug uptake through EPI-PNPs and EPI-MNPs, and to identify the uptake of NPs via caveolae-mediated endocytosis. In Caco-2 cell monolayers, EPI-PNPs and EPI-MNPs showed 2.45-and 3.17-fold higher permeability (P_app_) compared with the drug solution, respectively. In situ SPIP study revealed 5.23- and 5.93-fold increases in P_app_, respectively. Pharmacokinetic studies in rats demonstrated 4.7- and 5.57-fold higher oral bioavailability for EPI-PNPs and EPI-MNPs, respectively, compared with the drug solution. In addition, in vivo antitumor efficacy studies showed comparable tumor suppression effects among intravenous epirubicin and two oral NPs. EPI-MNPs had 1.18-fold higher bioavailability and better tumor suppression than EPI-PNP. The findings could have reflected the specific binding between mannose residues and lectins present in lymphoid and non-lymphoid organs. 

Etoposide was also encapsulated in PLGA-NPs with quercetin, a P-gp inhibitor, to enhance oral bioavailability [192]. When quercetin was co-loaded, the NPs could permeate deeper into the intestine, as visualized by confocal imaging. In addition, quercetin increased oral bioavailability in rats by 2.4-fold compared with NPs without quercetin and 3.84-fold compared with Etosid^®^ soft gelatin capsule. Thus, quercetin could inhibit P-gp efflux, thereby enhancing etoposide absorption and oral bioavailability.

Wang et al., prepared enoxaparin sodium–PLGA hybrid NPs (EPNs) to encapsulate doxorubicin hydrochloride [193]. The negative polymer of enoxaparin sodium and the cationic drug formed an electrostatic complex. In situ SPIP study revealed higher K_a_ and P_app_ of drug-loaded EPNs than those of drug solution in all segments of the rat intestine. An everted intestinal ring model study of the endocytosis mechanism of EPNs revealed the dominance of caveolae- and clathrin-dependent endocytosis pathways. More doxorubicin hydrochloride-loaded EPNs were internalized by Caco-2 cells than that of the drug solution, as visualized by confocal laser scanning microscopy, confirming the P-gp inhibitory effect of EPNs. Pharmacokinetic study in rats revealed increases of drug-loaded EPNs in C_max_ (5.23-fold), bioavailability (3.63-fold), and t_1/2_ (2.47-fold) compared to the drug solution. 

Another study reported the use of trimethyl chitosan (TMC)-CSKSSDYQC (CSK) peptide conjugates for the preparation of NPs loaded with gemcitabine, which is a nucleoside analog approved for the treatment of breast, pancreatic, and bladder cancer [46]. CSK was used as a ligand to target goblet cells, which are mucus-producing cells in the gastrointestinal tract. CSK conjugation can also induce NP uptake through caveolae- and clathrin-mediated endocytosis [194]. TMC-CSK-NPs increased P_app_ by 1.92- and 4.44-fold compared with TMC-NPs and drug solution, respectively, in an ex vivo permeation study using porcine intestine. Mechanistically, the uptake of NPs involved clathrin- and caveolae-mediated endocytosis, which was attributed to the effect of the CSK peptide. Pharmacokinetic study in rats revealed a higher absolute bioavailability for drug-loaded TMC-CSK-NPs (60.14%) compared with those of drug-loaded TMC-NPs (54.03%) and drug solution (9.86%). In tumor-bearing mice, the tumor growth rate of the drug-loaded TMC-CSK-NPs group was 5.1-fold and 3.3-fold lower than that of the non-treated and gemcitabine solution groups, respectively.

**Figure 6:**
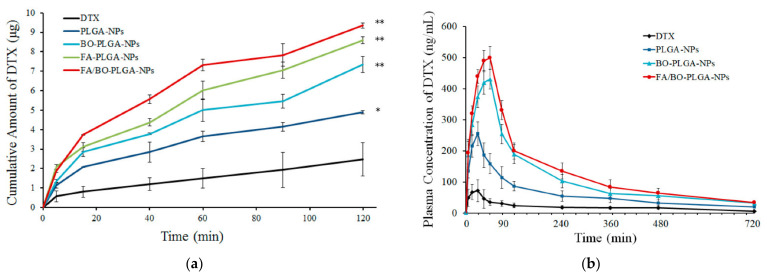
Effects of polymeric NPs on (**a**) intestinal absorption and (**b**) pharmacokinetic properties in rats of docetaxel (DTX). *p < 0.05, **p < 0.01. Reprinted from [195] under the terms of the Creative Commons Attribution License.

Yang et al., also prepared docetaxel-loaded PLGA-NPs, which were then coated with PEI-folic acid (FEI-FA) and PEI-borneol (PEI-BO) [195]. BO was used as a P-gp inhibitor to enhance drug absorption in the intestine, whereas FA induced folate receptor-mediated endocytosis of NPs. In the everted gut sac method, the NPs increased intestinal absorption by 6-fold compared to the docetaxel solution (Figure 6a). In addition, the NPs could be efficiently internalized into Caco-2 cells through folate receptor-mediated active targeting and BO-mediated P-gp inhibition. The bioavailability of FA/BO-PLGA-NPs was 6.8-fold higher than that of the drug solution, whereas the enhancements for BO-PLGA-NPs and PLGA-NPs were only 4.65- and 2.78-fold, respectively (Figure 6b).

Features of the aforementioned polymeric NPs are summarized in Table 5.

### 4.6. Other Pharmaceutical Formations for Inhibition of P-gp

In addition to the drug delivery system discussed above, other pharmaceutical formulations can also inhibit or bypass P-gp efflux and enhance drug absorption and oral bioavailability. These include microspheres, solid dispersions, and dendrimers. Microspheres are spherical microparticles 1–1000 μm in size that incorporate drugs within their core. Similar to polymeric NPs, microspheres can be prepared using various natural and synthetic polymers [196]. Thiolated xyloglucan was used to prepare microspheres loaded with lopinavir, which is an efficient drug for the treatment of HIV infection [197]. Lopinavir is a BCS class IV drug with low oral and variable bioavailability due to P-gp efflux and CYP3A4 metabolism. An ex vivo study using everted chick intestine revealed that the P_app_ of lopinavir was increased 3.15-fold after encapsulation in the microspheres. In addition, the relative bioavailability of the drug-loaded microspheres in rats was 3.22-fold higher than that of the free lopinavir and ritonavir dispersions. The enhancements were attributed to the absorption enhancing properties of the thiolated xyloglucan mucoadhesive polymer. 

In a recent study, a multifunctional nano-in-micro hierarchical microsphere system was developed using a co-flow microfluidic device [198]. Porous silica NPs were prepared to encapsulate meropenem, a last-resort antibiotic used to treat drug-resistant and acute infections. Then, the NPs were packed into different Eudragit^®^ polymeric microspheres. In Caco-2 cell monolayers, the microspheres prepared with Eudragit^®^ RSPO exhibited a 13.9-fold increase in P_app_ compared with the drug solution and a reduction in ER from 2.62 to 0.35. A decrease in TEER suggested the transient opening of tight junctions due to silica nanoparticles or microspheres, as well as the permeation of the drug across the cell monolayer through cellular endocytosis of NPs. The prepared microspheres retained the antibacterial activity of meropenem against *Staphylococcus aureus* and *Pseudomonas aeruginosa* by reducing the minimum inhibitory concentration that inhibits 90% of the target bacteria (MIC_90_) by 2-fold.

Solid dispersions are dispersions of drugs in solid matrices of polymers or small molecules. The dispersed state can be eutectic mixtures, amorphous/crystalline suspensions, or crystalline/glass solutions [199]. Gurunath et al., prepared a solid dispersion of candesartan with polyvinylpyrrolidone (PVP) K30 and naringin, a natural flavonoid P-gp inhibitor, using different methods that included kneading, spray drying, and freeze drying [200]. Candesartan is a selective angiotensin II receptor antagonist with low oral bioavailability (approximately 15% in humans) due to its low aqueous solubility and P-gp efflux in the gastrointestinal tract. Pharmacokinetic studies in rabbits showed that the incorporation of naringin into candesartan solid dispersions could increase oral bioavailability by 1.3- to 1.6-fold compared with candesartan solid dispersion. The optimized solid dispersion was freeze-dried solid dispersions with naringin, which produced a 3.7-fold increase in oral bioavailability compared to that of free candesartan suspension. Naringin, a P-gp inhibitor, effectively increased the intestinal permeability and oral bioavailability of a P-gp substrate.

Ticagrelor is an antagonist of the P2Y12 receptor, which is used as a platelet aggregation inhibitor. It is a BCS class IV drug with moderate intrinsic permeability and low solubility. Solid dispersion of ticagrelor was prepared with TPGS and Neusilin US2, a magnesium aluminometasilicate, using the ethanol solvent evaporation method [201]. In Caco-2 cell monolayers, P_app_ of the drug in solid dispersion increased by 1.4-fold, and ER decreased by 2.2-fold compared to the drug solution. The solid dispersion improved C_max_ by 2.38-fold and relative bioavailability by 2.20-fold compared with that of the drug suspension after oral administration in rats. TPGS was used as a carrier for the preparation of solid dispersions with the ability to enhance drug dissolution. In this study, it was also a P-gp inhibitor that increased absorption of the P-gp substrate. 

Zi et al., prepared solid dispersions of lopinavir with Kollidon VA 64 (a hydrophilic polymer) or Soluplus (a polymeric surfactant) using hot melt extrusion [128]. In situ permeability study revealed that Soluplus increased the permeability of lopinavir through the rat intestine by 1.6-fold without verapamil and 2.0-fold with verapamil (100 µM) compared with the drug alone. In Caco-2 cell monolayers, P_app (A-to-B)_ of lopinavir in a Soluplus solid dispersion was 2.23- and 1.50-fold higher than that in the cases of pure drug and pure drug + verapamil (100 µM), respectively. In addition, the oral bioavailability of lopinavir in Soluplus solid dispersion was 1.70-fold of a Kollidon VA 64 solid dispersion and 3.70-fold of the drug crystal, whereas the C_max_ was 1.61- and 3.64-fold higher, respectively. Soluplus matrixed extrudate could improve lopinavir bioavailability by hydrogen bonding with the drug, forming micelles in water, and by inhibiting P-gp. 

Dendrimers are tree-like branched structures that are used in various targeted drug delivery systems. They consist of an initial core, interior layers with repeating units, and an exterior with terminal functionality [202]. Poly(amido amine) (PAMAM) dendrimers have been extensively evaluated for the oral delivery of different small molecule drugs. They are both intestinal penetration enhancers and carriers for the transepithelial transport of drugs [203]. Camptothecin is a potent topoisomerase I inhibitor with antitumor and anti-HIV activities. The drug has poor and variable oral bioavailability due to its low solubility, low permeability, and P-gp efflux. Sadekar et al., developed different camptothecin-PAMAM dendrimer formulations, including cationic amine-terminated PAMAM dendrimer generation 4.0 (G4.0-NH_2_) and anionic carboxylate-terminated PAMAM generation 3.5 (G3.5-COOH) [204]. After oral administration to mice, the C_max_ at 2 h of ^3^H-camptothecin-PAMAM was increased 2–3-fold compared with ^3^H-camptothecin alone. PAMAM G4.0 (300 mg/kg) and G3.5 (1000 mg/kg) exhibited similar blood concentration–time profiles after oral administration. They did not increase mannitol transport, indicating the absence of paracellular transport via tight junction modulation. In addition, the liver concentration of the drug was similar between PAMAM G4.0 (300 mg/kg) and G3.5 (1000 mg/kg), and it was 2-fold higher than that of the drug alone. The epithelial layer of the small intestinal segments showed no histological evidence of toxicity. The findings indicate that PAMAM–drug complexes can bypass P-gp efflux and enhance drug absorption by endocytic uptake.

Ma et al., developed nanoliposomes modified with a PEGylated G5 PAMAM dendrimer (G5-PEG) for the oral delivery of probucol, which is an antioxidant drug commonly used to lower blood plasma cholesterol levels [205]. Probucol has poor aqueous solubility and low oral absorption efficiency due to P-gp efflux. In Caco-2 cell monolayers, the hybrid system increased P_app_ and decreased ER compared with those of probucol-loaded liposomes, indicating that modification with G5-PEG could effectively protect the drug from P-gp efflux. The authors also identified the uptake of liposomes and hybrid systems via caveolae-independent endocytosis pathways. In a hyperlipidemic mouse model, the hybrid systems showed higher lipid-lowering effects (plasma total cholesterol and triglyceride) compared with probucol suspension and a commercial probucol tablet. 

The aforementioned microspheres, solid dispersions, and dendrimers with P-gp inhibitory effects are summarized in Table 6.

## 5. Outlooks and Concluding Remarks

Oral administration is one of the most important routes of drug delivery. The presence of P-gp in the intestine plays a critical role in the active transport of many foreign substances back to the lumen and prevents them from entering the bloodstream [3]. The ability of P-gp to transport various substrates with different structures results in low permeability and poor oral bioavailability for many P-gp substrates. Various P-gp inhibitors have been synthesized and developed to inhibit P-gp to improve drug bioavailability and therapeutic effects [28,29,30]. Specific P-gp inhibitor drugs may result in drug–drug interactions and undesirable side effects due to their pharmacological activity. They are being replaced by new P-gp inhibitors with low or no pharmacological effects and improved potency and selectivity [107]. In addition, various natural bioactive constituents have been found to possess P-gp modulating effects [72]. Their activities can be further improved after modification by semi-synthesis. Pharmaceutical excipients with P-gp inhibitory effects have garnered much attention for many years [117,118]. They can modulate P-gp activity and produce no or low nonspecific pharmacological effects on the body. Furthermore, they are usually GRAS materials and frequently used in many drug delivery systems [60,105]. These drug delivery systems, including micelles, liposomes, emulsions, SMEDDS, SNEDDS, SLNs, NLCs, and polymeric NPs, can have inherent P-gp inhibitory effects attributed to their excipients. In addition, each drug delivery system, due to its structure, size, surface activity, and other components, may have various effects on bypassing P-gp. These effects include increased drug aqueous solubility, mucoadhesion, tight junction modulation, endocytosis, uptake by M cells, and lymphatic absorption via chylomicron uptake [135]. These dual advantages make pharmaceutical formulations a favorable approach to overcome the poor permeability induced by the P-gp efflux of many P-gp substrates. In addition, a P-gp inhibitor drug or natural constituent can be co-administered or co-delivered with pharmaceutical formulations to improve P-gp inhibition further [44,48,189]. To the best of our knowledge, none of the specific P-gp inhibitors has been successful in clinical trials [206,207]. The difficulties in clinical trials of P-gp inhibitors likely include toxicities (due to relatively high systemic concentrations of P-gp inhibitors to have an efficacy), drug interactions, and problems in clinical trial design [16]. Despite limited success in the clinical situation, P-gp inhibitors and their incorporation into pharmaceutical formulations are being investigated due to the relevance and importance of P-gp in drug discovery and development. Some pharmaceutical formulations of P-gp substrates are approved for oral administration, such as Sandimmune (cyclosporine A), Neoral (cyclosporine A), and Norvir (ritonavir) [208]. They may have P-gp inhibitory effects due to the presence of P-gp inhibitors (e.g., Cremophor RH40 in Neoral [209] and Cremophor EL in Norvir [210]) and bypass P-gp efflux by other mechanisms related to the formulation (SMEDDS). Due to the importance of oral drug delivery, it is expected that more pharmaceutical formulations would be available for the oral delivery of P-gp substrates, and their P-gp inhibitory effects would be clarified.

There are several considerations when developing a formulation of a P-gp substrate to bypass and inhibit P-gp efflux. First, when natural constituents are used as P-gp inhibitors, their pharmacokinetic properties, potential toxicity, and possible drug–drug interactions should be determined because they may have their own pharmacological activity. In addition, the optimal inhibitory concentrations for P-gp inhibition must be defined [116]. Second, the co-administration or co-delivery of P-gp inhibitor drugs and natural constituents with drug delivery systems containing P-gp substrates may or may not show beneficial effects. Beneficial effects can be observed in many cases, such as the co-administration of cyclosporine A and PTX-loaded SMEDDS (higher bioavailability) [49], co-encapsulation of cyclosporine A and docetaxel into SNEDDS (higher oral bioavailability) [48], and co-encapsulation of piperine and rapamycin in PLGA-NPs (higher permeability and oral bioavailability) [189]. In contrast, several cases reported no beneficial effects of co-administration of verapamil and andrographolide-loaded NLCs (no change in P_app_ in Caco-2 cell monolayers) [170] or co-administration of verapamil and tilmicosin-loaded NLCs (no change in P_app_ and ER in Caco-2 cell monolayers) [174]. Therefore, it is necessary to evaluate the effects of co-administration or co-delivery of P-gp inhibitor drugs and natural constituents with drug delivery systems, and to identify whether it is necessary or not. Third, the co-administration and co-delivery of P-gp inhibitor drugs and natural constituents with drug delivery systems of P-gp substrates have distinct advantages and disadvantages. Co-administration offers flexibility when P-gp inhibitors and formulations are combined. The dose ratios between them can be altered. If the initial P-gp inhibitor is not favorable to an individual, it can be replaced by another inhibitor. However, since the P-gp inhibitor is not encapsulated together with the P-gp substrates, their residence time in the gastrointestinal tract can be different, leading to a reduced P-gp inhibitory effect. Maintaining the effective inhibition of P-gp using P-gp inhibitors with low aqueous solubility, such as curcumin, is challenging when co-administered in the free form together with formulations of P-gp substrate. The co-encapsulation of a P-gp inhibitor and a P-gp substrate in the same drug delivery systems allows them to be presented together in the gastrointestinal tract. Therefore, P-gp inhibition and drug permeability can be maximized. However, the dose ratio between the P-gp inhibitor and P-gp substrate is fixed and cannot be changed, whereas replacement with other P-gp inhibitors is impossible. Therefore, the dose ratio and optimal P-gp inhibitor should be intensively investigated during formulation development. Fourth, oral absorption, bioavailability, and therapeutic effects of P-gp substrates are altered after encapsulation in pharmaceutical formulations with P-gp inhibitory properties. Different P-gp substrates are affected differently by P-gp efflux. Therefore, the dose of the P-gp substrates in the formulation should be adjusted. There is a need to establish the relationship between the P-gp inhibitor dose, P-gp substrate dose in the formulations, and oral bioavailability or therapeutic activity [60]. There are also other concerns related to pharmaceutical formulations, such as toxicity and long-term side effects of synthetic nanomaterials and batch-to-batch inconsistency, which limit the production of many nanocarriers on a large scale. 

A large number of drugs have been identified as P-gp substrates. Thus, the development of pharmaceutical formulations with P-gp inhibitory effects for the oral delivery of these drugs is an essential and inevitable task of formulators [29]. Many drug delivery systems have been proven to possess inherent P-gp inhibitory effects and the ability to bypass P-gp efflux via different mechanisms. Therefore, the encapsulation of P-gp substrate into these drug delivery systems will be continued with a particular focus on the utilization of various pharmaceutically inert excipients [60,105]. New excipients with good stability and low toxicity can be developed to provide better P-gp inhibitory effects. In addition, in vitro–in vivo correlation can be established, which will be helpful in predicting the in vivo properties of the formulations from in vitro behaviors, although this task is very challenging. Some studies may be conducted to clarify the contribution of P-gp inhibitors and formulations in the overall effects on increasing the oral absorption and bioavailability of P-gp substrates; however, as oral absorption is a complex process, it is difficult to distinguish the effects of each component in vivo. Natural constituents are promising P-gp inhibitors. They can be evaluated for their optimum inhibitory concentration, pharmacokinetic properties, toxicity, and drug–drug interactions prior to application [116]. Finally, the inhibition and bypassing mechanisms of the formulations will be intensively studied to provide the basis for designing formulations of P-gp substrates.

## Figures and Tables

**Table 1:** Classification of P-glycoprotein inhibitors.

Classification		Examples
Small molecules	First generation [109]	Verapamil, cyclosporine, trifluoperazine, quinidine, reserpine, yohimbine, tamoxifen, toremifene, and vincristine
	Second generation [110,111]	Dexverapamil and PSC 833 (valspodar)
	Third generation [107]	VX-710 (biricodar), GF120918 (elacridar), LY335979 (zosuquidar), XR9576 (tariquidar), R101933 (laniquidar), WK-X-34, and OC144-093 (ontogeny)
Natural products [72]	Alkaloids	Glaucine, pervilleine, berberine, kopsiflorine, lobeline, cepharanthine, ibogaine, theobromine
	Flavonoids	Quercetin, morin, phloretin, rhamnetin, plagiochin E, daidzin, procyanidine, rotenone
	Coumarins	Decursinol, bergaptol, galbanic acid, farnesiferol
	Terpenoid	Citral, latilagascene, paraliane, pepluanin A, jolkinol B, euphoportlandol lhelioscopinolide, tuckeyanols, euphotuckeyanol, isopimaric acid, totarol
	Saponins	Gracillin, tenacissimoside A, karavilagenin C, balsaminol, ginsenoside F1, protopanaxatriol
	Peptides	Discodermolide, kendarimide, hapalosin, nocardioazine
	Resins	Gambogic acid, orizabin
	Miscellaneous natural compounds	Acetoxy cavicolacetate, arctigenin, pheophorbide, porphyrin, cannabinol, gomisin, pregomisin, phenylbutanoid
Pharmaceutical excipients	Surfactants [40,60]	Polysorbates: polysorbate 80, 20 Sucrose esters: sucrose monolaurate Tocopheryl ester: TPGS PEG esters: Cremophor EL, Solutol HS-15, Labrasol, Softigen 767, Myrj 52, Gelucire 44/14 PEG ethers: Brij 78 Other: sodium 1,4-bis (2-ethylhexoxy)-1,4-dioxobutane-2-sulfonate (AOT), cetyltrime-thylammonium bromide (CTAB)
	Polymers [60,123,124]	Natural polymers: dextrans, agar, gellan gum, gum arabic, gum traganth, guar gum, carrageenan gum, xanthan gum, alginates, chitosan Amphiphilic diblock copolymers: methoxyPEG-block-polycaprolactone (MePEG-b-PCL), Soluplus Pluronic block copolymers: poloxamer 407 and 188
	Others [129,130,131,132]	Glycerides: monoolein (Peceol^TM^), monostearin Phospholipids: 8:0 PC, 10:0 PC Methylated cyclodextrin

TPGS: α-tocopheryl polyethylene glycol-1000-succinate, PEG: polyethylene glycol.

**Table 2:** SMEDDS, SNEDDS, emulsions, and liposomes with P-gp inhibitory effects.

Drug(P-gp Substrate)	Formulation	Components	Major Outcome	Ref.
Paeonol	Emulsion	Isopropyl myristate, Cremophor EL35 *, ethanol	- Caco-2 cells monolayers: increase in P_app (A-to-B)_ (1.7-fold) and decrease in ER (1.7-fold) compared with drug solution. - SPIP: increases in P_app_ (~1.5-fold) in ileum and colon compared with drug solution. - Everted gut sacs of rats: increases in P_app_ (~2 and 1.5-fold) compared with drug solution and drug in Cremophor EL35 solution, respectively. - In vivo pharmacokinetic study in rats: increases in oral bioavailability (4.27-fold) and C_max_ (4.02-fold) compared with drug suspension.	[79]
Etoposide	SMEDDS	Ethyl oleate, medium-chain triglyceride, Cremophor RH40 *, Cremophor EL *, polysorbate 80 *	- SPIP: increases in P_app_ (2.7-fold for polysorbate 80-based SMEDDS and ~1.7-fold for two other SMEDDS). - In vivo pharmacokinetic study in rats: increases in oral bioavailability (1.4, 1.7, and 2.5-fold for Cremophor RH40-, Cremophor EL-, and polysorbate 80-based SMEDDS, respectively) compared with drug suspension.	[47]
Irinotecan	SMEDDS	Capmul MCM-C8, Cremophor EL *, Pluronic L-121*	- Caco-2 cells: increased penetration to the intestine and uptake of fluorescent probe. - In vivo pharmacokinetic study in rats: increases in oral bioavailability (4.29-fold) and C_max_ (1.77-fold) compared with drug suspension.	[52]
Candesartan cilexetil	SNEDDS	Peppermint oil, Cremophor RH40*, Labrasol *	- Everted gut sacs of rats: increase in P_app (A-to-B)_ (~2-fold) and decrease in ER (3.52-fold) compared with drug solution. - Pharmacokinetic study in rabbits: increases in oral bioavailability (1.69-fold) and C_max_ (1.75-fold) compared with ATACAND tablets.	[53]
Docetaxel	SNEDDS	Capryol 90, Cremophor EL *, Transcutol HP, cyclosporine A *	- SPIP: increases K_a_ and P_app_ (≈1.5-fold for docetaxel-loaded SNEDDs and ≈2-fold for docetaxel–cyclosporine A co-loaded SNEDDS) compared with drug solution. - In vivo pharmacokinetic study in rats: increase oral bioavailability (2.7-fold for docetaxel-loaded SNEDDS and 9.2-fold for docetaxel–cyclosporine A co-loaded SNEDDS) compared with drug solution. - In vivo antitumor efficacy in mice: increase antitumor efficiency for both SNEDDS formulations compared with drug solution (oral administration).	[48]
Valsartan	Supersaturable SMEDDS	Capmul MCM, Transcutol P, Poloxamer 407 *, polysorbate 20 *, polysorbate 80 *, Cremophor EL *, Labrasol *	- SPIP: increase in P_eff_ (2.3–4.1-fold) compared with drug solution. - In vivo pharmacokinetic study in rats: increases in oral bioavailability (2.62–4.70-fold) and C_max_ (3.65–5.94-fold) compared with drug suspension.	[54]
PTX	SMEDDS	Captex 355 (caprylic/capric triglyceride), Cremophor EL *, polysorbate 80 *, PEG 400	- In vivo pharmacokinetic study in rabbits: increase in oral bioavailability (4.55-fold) compared with PTX suspension; further increase in oral bioavailability when pretreatment with 2 doses of cyclosporine A.	[49]
Docetaxel	Nanoliposomes coated with folate grafted thiolated chitosan	Dipalmitoylphosphatdly choline, phosphatidylcholine, cholestrerol, oleic acid, folate grafted thiolated chitosan *	- Everted gut sac of rats: increases in P_app (A-to-B)_ (9.62 and 13.12), decreases in ER (from 5.78 to 2.36 and 1.0) for nanoliposomes and thiomer enveloped nanoliposomes, respectively. - In vivo pharmacokinetic study in rabbits: increases in oral bioavailability (6.2 and 13.6-fold), C_max_ (4.2 and 10.3-fold), and t_1/2_ (from 33 h to 72 and 86 h) for nanoliposomes and thiomer enveloped nanoliposomes, respectively, compared with drug suspension.	[162]
PTX	Liposome–micelle hybrid (micelles in liposomes)	Pluronic F127 *–polyethylenimine copolymer, sodium cholate, soybean phosphatidylcholine	- SPIP: increase in P_eff_ (12.8-fold) compared with PTX solution. - In vivo pharmacokinetic study in rats: increases in oral bioavailability (4.33-fold) and C_max_ (≈3.3-fold) compared with PTX solution.	[90]
Lovastatin	Liposome–micelle hybrid (micelles in liposomes)	Phosphatidylcholine *, cholesterol, 1,2-distearoyl-sn-glycero-3-phospho-1′-rac-glycerol sodium-salt, TPGS*	- Caco-2 cell monolayers: increase in lovastatin absorption (1.14-fold), transportation (2-fold), and cellular uptake (2.88-fold) compared with free drug.	[55]

*: P-gp inhibitor, PTX: paclitaxel, SMEDDS: self-microemulsifying drug delivery systems, SNEDDS: self-nanoemulsifying drug delivery systems, C_max_: maximum concentration in blood, P_app (A-to-B)_: apparent permeability in the apical-to-basolateral direction, ER: efflux ratio, SPIP: single-pass intestinal perfusion, P_app_: apparent permeability coefficient, K_a_: absorption rate, t_1/2_: half-life, P_eff_: effective permeability coefficient.

**Table 3:** SLNs and NLCs with P-gp inhibitory effects.

Drug (P-gp Substrate)	Components	Major Outcome	Ref.
Andrographolide	Compritol ATO888, lecithin, glycerylmonostearate, Tween 80 *	- Caco-2 cell monolayers: increase in P_app (A-to-B)_ (2-fold) and decrease in ER (4.13-fold) compared with drug solution; no change in P_app_ values when altering temperature (37 and 4 °C) or adding verapamil. - In vivo pharmacokinetic in rats: increases in bioavailability (2.41-fold) and C_max_ (3.63-fold) compared with drug suspension.	[170]
Curcumin	Glyceryl monostearate, soya lecithin, Brij 78 *, TPGS *	- SPIP: increase in P_eff_ (1.64-fold for SLNs, 1.28-fold for Brij 78 solution, 1.39-fold for Brij 78-TPGS solution) compared with drug solution. - In vivo pharmacokinetic in rats: increases in bioavailability (9.42-fold), C_max_ (3.54-fold), T_max_ (from 0.58 to 5.71 h), and MRT (3.59-fold) compared with drug suspension.	[171]
Lumefantrine	Stearic acid, caprylic acid, TPGS *, poloxamer 188 *	- SPIP: increases in P_eff_ (3-fold) and K_a_ (2.96-fold) compared with drug solution. - In vivo pharmacokinetic in mice: increases in bioavailability (2.2-fold) and C_max_ (2.7-fold) compared with drug suspension.	[173]
Mangiferin	Phospholipon 90 G, Compritol 888 ATO, Labrafil M 2125, Tween 80 *, soy lecithin *	- Caco-2 cell monolayers: increase in P_app (A-to-B)_ (9.4 for PLCs and 10.1-fold for PLC-NLCs) compared with drug solution. - SPIP: increases in P_app_ of PLCs and PLCs-NLCs compared with drug solution and drug + verapamil solution. - In vivo pharmacokinetic study in rats: increases in bioavailability (2.1-fold) and C_max_ (4.8-fold) compared with drug suspension.	[86]
Aripiprazole	Tristearin, sodium taurocholate, Tween 80 *	- In vivo pharmacokinetic in rats: increases in bioavailability (1.6-fold) and C_max_ (2-fold) compared with drug suspension.	[50]
Darunavir	Hydrogenated castor oil, sodium oleate *	- Caco-2 cell monolayers: increase in P_app_ (4.04-fold) compared with drug solution. - SPIP: increases in AUC (2.50-fold in stomach and 2.51-fold in duodenum) compared with drug solution. - In vivo pharmacokinetic study in rats: increases C_max_ (2.61–2.69-fold) and bioavailability (4.81–5.69-fold) compared with drug suspension. - In vivo biodistribution: increase drug accumulation in the brain (1.5–3.5-fold) compared with drug suspension.	[91]
Tilmicosin	Carnauba wax, PVA, Tween 80 *	- SPIP: increases in P_app_ (2.08-fold) and K_a_ (1.67-fold) compared with drug solution; increases in P_app_ (2.32-fold) and K_a_ (2.13-fold) when adding 1% Tween 80. - In vivo pharmacokinetic study in pigs: enteric granules containing SLNs increased bioavailability (2.72-fold) and C_max_ (2.92-fold) compared with a commercial premix.	[89]
Tilmicosin	Stearic acid, oleic acid, Tween 80 *	- Caco-2 cell monolayers: increase in P_app (A-to-B)_ (1.84-fold) and decrease in ER (2.64-fold) compared with drug solution; no change in P_app_ and ER values when adding verapamil. - In vivo pharmacokinetic in rats: increase in bioavailability (1.42-fold) compared with drug suspension.	[174]
Tilmicosin	Stearic acid, palmitic acid, lauric acid, oleic acid, Tween 80 *	- Caco-2 cell monolayers: increase in P_app (A-to-B)_ (1.69–2.03-fold) and decrease in ER (1.22–1.47-fold) compared with drug solution. - In vivo pharmacokinetic in chickens: increases in bioavailability (2.04-fold) and C_max_ (1.36) of drug-loaded stearic acid-NLCs compared with drug suspension.	[175]
Raloxifene	Glyceryl monostearate, Compritol 888 ATO, TPGS-1000 *, Phospholipid S-100	- Caco-2 cells: increase cellular uptake (3.18-fold). - P-gp efflux study: decrease in percentage of cells showing efflux. - In vivo pharmacokinetic in rats: increases in bioavailability (4.56-fold), C_max_ (4.06), and t_1/2_ (2.12-fold) compared with drug suspension.	[87]
Linagliptin	Palmitic acid, poloxamer 188 * and Tween 80 *	- In vitro: increase in permeability compared with drug solution (1.74, 1.82, and 1.76-fold in Caco-2 cell, SPIP, and everted gut sac permeability studies, respectively). - In vivo: Increased oral bioavailability in rats (3-fold), lower blood glucose levels in rats.	[43]
PTX	Glyceryl monooleate, Soluplus, chitosan, TPGS *, pluronic P123 *, Solutol HS15 *	- SPIP: increase in P_app_ value (2.46-fold in duodenum, 1.35-fold in ileum for TPGS-incorporated PLHNs) compared with Taxol. - In vivo pharmacokinetic study in rats: increase in bioavailability (6.91–7.51-fold) compared with Taxol.	[126]

*: P-gp inhibitor, SLNs: solid lipid nanoparticles, NLCs: nanostructured lipid carriers, T_max_: time to reach C_max_, MRT: mean residence time, PLCs: phospholipid complex, AUC: area under the curve, PLHNs: polymer–lipid hybrid nanoparticles.

**Table 4:** Micelles with P-gp inhibitory effects.

Drug (P-gp Substrate)	Components	Major Outcome	Ref.
PTX	Bromotetrandrine (W198) *, TPGS *, Solutol HS15 *	- Caco-2 cells: increase in permeability (2.45-fold) compared with Taxol. - In vivo pharmacokinetic study in rats: increases in oral bioavailability (2-fold) and C_max_ (2-fold) compared with Taxol. - Antitumor efficacy on xenografts mice: higher tumor inhibition rate than Taxol; similar tumor inhibition rate between oral and intravenous administration of the micelles.	[133]
PTX	Bromotetrandrine (W198) *, Tween 80 *, Cremophor EL *	- Comparing W198/PTX micelles with PTX micelles: higher cellular uptake (10-fold) in Caco-2 cells; increases in bioavailability (5.7-fold) and C_max_ (2.8-fold) in rats.	[28]
PTX	PEG17-b-PCL5	- MDCKII and MDCK-MDR1 cells: increase in cellular uptake of both P-gp (PTX, Rhodamine 123) and non-P-gp (coumarin) substrates. - MDCK-MDR1 cell monolayers: increase in P_app_ of Rhodamine 123 (1.62-fold) compared with Rhodamine 123 solution. - In vivo pharmacokinetic study in rats: increases in C_max_ (3.61-fold) and oral bioavailability (2.61-fold) compared with PEG114-b-PCL88 micelles.	[82]
PTX	OPPC *	- In vivo fluorescence imaging study: enhanced stability and integrity of mouse gastrointestinal tract. - Caco-2 cells: increase in transport and accumulation. - In vivo pharmacokinetic study in rats: increase in oral bioavailability (5.5, 3.8, and 1.3-fold higher than Taxol, Taxol + verapamil, and PTX-loaded OPPC micelles via duodenum, respectively).	[179]
PTX	Carboxymethyl chitosan-rhein	- Caco-2 cells: the micelles were taken up into the enterocyte as whole. - SPIP: increase in absorption in the duodenum, jejunum, ileum, and colon compared to Taxol, Taxol + verapamil, and Taxol + carboxymethyl chitosan-rhein conjugate without notable injury to intestinal villi.	[134]
PTX	Gallic acid-chitosan-TPGS * copolymer	- Caco-2 cells: increase in permeability compared with PTX suspension. - In vivo pharmacokinetic study in rats: increases in oral bioavailability (3.80-fold) and C_max_ (3.95-fold) compared with PTX suspension. - Increase in anti-lung tumor efficacy compared with Taxol in mice.	[137]
DBAE	Soluplus, TPGS *	- Caco-2 cells: increase in cellular uptake (2–2.6-fold) compared with DBAE suspension. - Intestinal absorption in rats: 8 and 5-fold increases (in ileum at 2 h and 4 h, respectively). - In vivo pharmacokinetic study in rats: increases in oral bioavailability (3.37-fold) and C_max_ (2.92-fold) compared with DBAE suspension. - Increase in thrombosis inhibition rate (4.24-fold) compared with DBAE suspension	[127]
Cyclosporine A	Curcumin *–carboxymethyl chitosan and low-molecular-weight heparin–all-trans-retinoid acid conjugate	- SPIP: increase in intestinal absorption compared with the drug suspension and mixture of drug and curcumin-carboxymethyl chitosan in the duodenum and jejunum segments. - In vivo pharmacokinetic study in rats: increases in oral bioavailability (6.02-fold) and C_max_ (15.13-fold) compared with drug suspension.	[44]
Doxorubicin	Lysine-linked ditocopherol PEG 2000 succinate (PLV_2K_)	- SPIP: increases in K_a_ (3.19, 1.61, and 1.80-fold in duodenum, jejunum, and ileum, respectively). - In vivo pharmacokinetic study in rats: increase in oral bioavailability (5.6-fold) compared with drug solution.	[183]
Berberine	Tween 80 *, Pluronic 85 *	- Caco-2 cell monolayers: increase in permeability (3.64-fold) and decrease in the ER (from 7.5 to 1.1). - In vivo pharmacokinetic study in rats: increase in oral bioavailability (15.6-fold) compared with berberine suspension.	[45]
Aripiprazole	Soluplus, TPGS *	- Parallel artificial membrane permeability assay for intestine: increase in permeability (2-fold) compared with free drug. - In vivo pharmacokinetic study in rats: increases in oral bioavailability (1.60-fold) and C_max_ (1.64-fold).	[184]

*: P-gp inhibitor, DBAE: dabigatran etexilate, MDCK: Madin–Darby canine kidney, MDR: multidrug resistance, PCL: poly(ε-caprolactone), OPPC: *N*-octyl-*N*’-phthalyl-O-phosphoryl chitosan.

**Table 5:** Polymeric nanoparticles with P-gp inhibitory effects.

Drug (P-gp Substrate)	Polymer	Major Outcome	Reference
PTX	Poly(methyl vinyl ether-co-maleic anhydride), PEGs	- Ex vivo permeability studies: increases in P_app_ (1.8-fold for uncoated and 3.3–7.3-fold for PEG-coated NPs). - In vivo pharmacokinetic study in rats: increases in absolute oral bioavailability (1.7–7.7-fold) compared with uncoated NPs.	[188]
Tacrolimus	CD-PVM/MA amphiphilic copolymer	- In vivo pharmacokinetic study in rats: increase in bioavailability (9.51-fold) and C_max_ (11.54-fold) compared with drug solution.	[104]
Rapamycin	PLGA	- Everted gut sac: NPs increased permeability 5-fold after co-encapsulated piperine. - In vivo pharmacokinetic study in rats: increases in bioavailability (3.5 and 4.8-fold for NPs without and with piperine co-encapsulation, respectively) compared with drug suspension.	[189]
Rapamycin	TPGS, lecithin, zein	- Caco-2 cell: increase cellular uptake. - Caco-2 cell monolayers: increase in P_app_ (1.39-fold) and decrease in ER (from 1.47 to 1.02). - SPIP: increases in K_a_ and P_eff_ in all segments of rat intestine. - In vivo pharmacokinetic study in rats: increases in bioavailability (2.64-fold) and C_max_ (2.04-fold) compared with drug solution.	[190]
Epirubicin	PLGA, PEG, mannosamine	- Caco-2 cell: increases in cellular uptake and P_app_ (2.45-fold for EPI-PNPs and 3.17-fold for EPI-MNPs) compared with drug solution. - SPIP: increases in P_app_ (5.23-fold for EPI-PNPs and 5.93-fold for EPI-MNPs) compared with drug solution. - In vivo pharmacokinetic study in rats: increases in bioavailability (4.7-fold for EPI-PNPs and 5.57-fold for EPI-MNPs) compared with drug solution. - In vivo antitumor: comparable tumor suppression effect among intravenous epirubicin and two oral NPs.	[191]
Etoposide	PLGA	- In vivo pharmacokinetic study in rats: etoposide–quercetin co-loaded NPs increased bioavailability 2.4 and 3.84-fold compared with etoposide-loaded NPs and Etosid^®^, respectively.	[192]
Doxorubicin	Enoxaparin sodium, PLGA	- SPIP: increases in K_a_ and P_app_ compared with drug solution. - Caco-2 cells: increase in drug uptake. - In vivo pharmacokinetic study in rats: increases in C_max_ (5.23-fold), bioavailability (3.63-fold), and t_1/2_ (2.47-fold) compared with drug solution.	[193]
Gemcitabine	TMC-CSKSSDYQC peptide conjugates	- Ex vivo permeation: increases in P_app_ (1.92 and 4.44-fold) compared with TMC-NPs and drug solution, respectively. - In vivo pharmacokinetic study in rats: increases in absolute bioavailability (60.14%) compared with TMC-NPs (54.03%) and drug solution (9.86%). - In vivo antitumor efficacy: decreases in tumor growth rate (5.1 and 3.3-fold) compared with non-treated and drug solution groups.	[46]
Docetaxel	PLGA, PEI-FA, PEI-BO	- Everted gut sac: increase in P_app_ (6-fold) compared with drug solution. - Caco-2 cells: increase in drug uptake. - In vivo pharmacokinetic study in rats: increases in bioavailability (6.8, 4.65, and 2.78-fold for FA/BO-PLGA-NPs, BO-PLGA-NPs, and PLGA-NPs, respectively) compared drug solution.	[195]

PLGA: polyglycolic acid copolymers, CD-PVM/MA: poly(methyl vinyl ether-co-maleic anhydride)-graft-hydroxypropyl-β-cyclodextrin amphiphilic copolymer, TMC: trimethyl chitosan, PEI polyethylenimine, FA: folic acid, BO: borneol.

**Table 6:** Microspheres, solid dispersions, and dendrimers with P-gp inhibitory effects.

Drug (P-gp Substrate)	Formulation	Components	Major Outcome	Reference
Lopinavir	Microspheres	Thiolated xyloglucan	- Everted gut sac (chicken): increase in P_app_ (3.15-fold). - In vivo pharmacokinetic study in rats: increase in bioavailability (3.22-fold) compared with free lopinavir + ritonavir dispersion.	[197]
Meropenem	NPs in microspheres	Silica, Eudragit^®^ RSPO	- Caco-2 cell monolayers: increase in P_app_ (13.9-fold) and decrease in ER (from 2.62 to 0.35). - Antibacterial activity: decreases in MIC_90_ (2-fold) in *Staphylococcus aureus* and *Pseudomonas aeruginosa* compared with drug solution.	[198]
Candesartan	Solid dispersion	PVP K30, naringin *	- In vivo pharmacokinetic study in rabbits: increases in bioavailability (3.7 and 1.3–1.6-fold) compared with candesartan solid dispersion (without naringin) and candesartan suspension, respectively.	[200]
Ticagrelor	Solid dispersion	TPGS*, Neusilin US2	- Caco-2 cell monolayers: increase in P_app_ (1.4-fold) and decrease in ER (2.2-fold) compared with drug solution. - In vivo pharmacokinetic study in rats: increases in bioavailability (2.20-fold) and C_max_ (2.38-fold) compared with drug suspension.	[201]
Lopinavir	Solid dispersion	Soluplus *	- SPIP: increase in permeability (1.6-fold). - Caco-2 cell monolayers: increase in P_app (A-to-B)_ (2.23-fold). - In vivo pharmacokinetic study in rats: increases in bioavailability (3.70-fold) and C_max_ (3.64-fold) compared with drug crystal.	[128]
Camptothecin	Dendrimers	PAMAM G3.5-COOH and G4.0-NH_2_	- In vivo pharmacokinetic study in mice: increases in C_max_ (2–3-fold) and drug concentration in liver (2-fold) compared with drug suspension.	[203]
Probucol	Dendrimers	PEGylated G5 PAMAM	- Caco-2 cell monolayers: dendrimer–nanoliposomes hybrid systems increased P_app_ and decreased ER compared with nanoliposomes. - Pharmacodynamic studies: higher lipid-lowering effects compared with drug suspension and a commercial tablet.	[205]

*: P-gp inhibitor, NPs: nanoparticles, MIC_90_: minimum inhibitory concentration that inhibits 90% of the target bacteria, PVP: polyvinylpyrrolidone, PAMAM: poly(amido amine).

## Data Availability

The data presented in this study are available in this article.
